# Market intelligence for exporting agricultural products to foreign markets: A PRISMA-based bibliometric and systematic review

**DOI:** 10.12688/f1000research.175062.2

**Published:** 2026-08-21

**Authors:** Antony Altamirano-Gonzales, Rogger Orlando Morán-Santamaría

**Affiliations:** 1 Administración y Negocios Internacionales, Universidad Cesar Vallejo, Trujillo, La Libertad, Peru

**Keywords:** agricultural exports; agri-food trade; market intelligence; competitive intelligence; export performance; agri-food value chains; bibliometric analysis; PRISMA; Latin America.

## Abstract

**Background:**

International trade in agri-food products faces growing challenges linked to price volatility, trade barriers and information asymmetries, factors that reduce the competitiveness of exporting companies. In this scenario, market intelligence (MI) is positioned as a fundamental tool for strengthening international marketing processes, as it enables the systematic collection and analysis of information to support strategic decision-making.

**Methods:**

This study employs a cross-sectional, descriptive, and non-experimental design based on a bibliometric and systematic analysis of the literature. A mixed methodology was used, integrating qualitative content synthesis with bibliometric indicators.

**Results:**

The results show that scientific production related to market intelligence has experienced particularly notable growth since 2018, with a predominant participation of countries such as the United States, China and the United Kingdom. The analysis identified a three-part structure consisting of the conceptual foundations of MI, its strategic applications in the field of agro-exports, and its documented effects on export performance. The evidence reviewed confirms that MI contributes to strengthening international competitiveness by reducing uncertainty and guiding strategic decision-making within companies.

**Conclusions:**

Despite the benefits associated with the use of market intelligence in the agro-export environment, its adoption remains limited, especially in Latin America, where technological and institutional gaps persist that hinder its full implementation. The available evidence suggests that MI is a strategic resource for improving export performance, although significant efforts are still required to expand its use and consolidate its contribution to the competitive development of the sector.

## 1. Introduction

International trade in agricultural goods is very important to the world economy. The expected value of the world’s trade in food and agricultural products in 2023 was USD 1.9 trillion, and it is still going up. ^1^ This is because of globalisation and the growing integration of food markets. Also, trade in agricultural goods is important for the economic growth of many countries and the safety of the world’s food supply. ^2^ In addition to making sure that food supplies are available in places where local production isn’t enough, it encourages specialisation and makes production more efficient around the world. ^3^ It also helps exporting countries reach bigger markets, diversify their economies, and make foreign currency, all of which encourage investment in new farming technologies and methods. ^4^ The growth of agricultural trade flows has a multiplier effect that increases business activity in the industry, strengthens value chains, and creates wealth. ^5^ But this dynamism also causes big problems, like price swings, tariffs and non-tariff barriers, phytosanitary rules, and logistical bottlenecks. These problems make it hard for exporting countries to keep their incomes stable and make it harder for manufacturers to compete. ^6^

Because of this, market intelligence (MI) has become a structured way to collect, analyse, and process important data to help businesses make decisions. Its importance is especially clear in international trade. For example, Donthu et al. (2021), ^7^ show how bibliometric approaches and data analysis methodological tools, which were also used in this work, can help find new trends and groups of related ideas in a certain topic. In the same way, Qorri and Felföldi (2024) ^8^ say that MI helps agricultural cooperatives respond to changes in global demand faster and better by reducing information asymmetries. These contributions not only define MI, but they also show how it works and how it can help manage the agri-food market in the early stages.

In international trade, it is becoming clearer and clearer how important it is to know what customers want, what quality and safety standards are, how to prepare for changes in regulations, and how to deal with competition from around the world. The scientific literature has predominantly examined these facets in a disjointed manner, scattered throughout agricultural economics, marketing, and development studies, despite a rising interest in MI and an increasing body of empirical evidence supporting its benefits. ^9^ This dispersion makes it hard to get a clear picture of how MI affects agricultural exports and the performance of exporting companies. It also stresses the need for research that takes all of these points of view into account.

Existing research provides illustrative empirical evidence. In exporting companies, MI has been associated with better performance through customer orientation and reduced uncertainty. ^10^ In India, wheat producers with better access to market information have achieved higher participation of small farmers and increased sales volumes ^11^; Methodologically, scales have been developed to measure farmers’ knowledge and use of MI, which supports price forecasting and more informed decision making. ^12^ Case studies in horticulture in Kenya and agri-food trade in Peru show that, when MI is combined with training and compliance with phytosanitary standards, it helps to open international markets, increase export volumes and improve profitability. ^13, 14^ These real-world experiences support the notion of MI as a system that creates value from data and helps to bridge the gap between theory and practice.

Beyond conceptual developments, theories of digital transformation have reframed MI as a technological capability. Ospina Usaquén et al. (2020) ^15^ note that the expansion of big data, machine learning and interactive dashboards has transformed analytical platforms, generating more sophisticated and precise environments for decision-making. This transformation not only improves efficiency, but also changes the ways in which firms adapt to complex, automated and data-driven systems. To organise this complexity and clarify how its components interact, Hernández Cruz et al. ^16^ propose a five-layer MI model (collection, processing, analysis, dissemination and application). This model explains the process, links each step to specific data sources, and stresses how important it is to use clear methods to lower information asymmetry and predict changes in demand. In this method, MI changes from a set of tasks into a strategic capacity that is powered by technology.

Despite these theoretical and empirical advancements, the application of MI to the global marketing of agricultural products and its impact on the sector’s competitiveness and sustainability in a globalised context remain unresolved. ^17^ This fragmentation hampers the development of an integrated perspective on the potential of MI to improve decision-making, reduce information asymmetries and optimise export performance.

This study therefore arises from the need to systematise existing knowledge, identify trends, methodologies and gaps in the scientific literature, and provide a consolidated view of MI as a strategic mechanism in the internationalisation of agro-exports. The aim is twofold: first, to contribute to the theoretical understanding of MI in the field of agricultural exports by integrating conceptual approaches; and second, to offer a practical basis for future research and sectoral policies. To that end, a systematic and bibliometric review of the scientific literature was conducted to characterise the evolution, main features and academic contributions of this body of work.

In light of this context, the study addresses the following general research question: to what extent does the existing scientific literature report that the implementation of MI by agricultural export firms contributes to improving their competitiveness in international markets? This overarching question is specified in three more focused research questions: (i) what is the current state of the literature on the relationship between MI and the international marketing of agricultural products? (ii) how have empirical studies analysed the relationship between MI and the marketing and export performance of agricultural products in foreign markets? and (iii) to what extent are there convergences in the empirical results reported by researchers regarding MI and agricultural exports?.

Consistent with these questions, the general objective of this study is to provide an integrated overview of how MI has been conceptualised and applied in the international marketing of agricultural products and of the empirical evidence linking MI to export performance, based on a bibliometric and systematic review of the scientific literature. The specific objectives are: (i) to identify the current state of the literature on the relationship between MI and the international marketing of agricultural products; (ii) to examine how empirical studies have operationalised and measured the relationship between MI and the performance of agricultural exports in foreign markets; and (iii) to identify convergences and gaps in the empirical results reported in this field.

## 2. Materials and methods

The methodology delineates the approach for reviewing and analysing the scientific literature regarding the application of market intelligence (MI) in the marketing of agricultural products internationally. This study employs a cross-sectional, descriptive, non-experimental design, grounded in a bibliometric and systematic analysis of the literature. A mixed methodology was employed, integrating qualitative content synthesis with quantitative bibliometric indicators. ^18^ This method was chosen because it fit with the study’s goals, which included making a systematic way to sort and understand research trends found in the current literature. The data was collected from high-impact academic databases, and the papers were selected based on their authorship, subject matter, year of publication, and relevance to the study issue. ^19^ This decision aligns with prior research recommendations, which underscore the importance of formulating precise research questions and objectives, executing a comprehensive search in credible scientific repositories, and establishing a rigorous selection, coding, and analysis protocol to ensure the validity of results in such reviews. ^20^

The Web of Science (WoS), Dimensions, and Scopus databases were selected for this purpose due to their comprehensive interdisciplinary coverage in agricultural economics, management, and marketing. These databases are often used in bibliometric studies because they have strict rules for indexing and quality control. They can also create standard metadata that research tools like Bibliometrix and VOSviewer can use. By combining these three sources of information—a curated index, a full academic index, and a database with a lot of information on current and regional publications—it makes it easier to find information and reduces selection bias. Reviews that want traceability and depth in metadata management often recommend this method. ^21^

Bibliometrics is an approach that uses statistical indicators from academic databases to look at how scientific work is used, how often it is cited, and how it is shown visually through co-occurrence maps and cooperation networks. ^22^ Bibliometric analysis has been demonstrated to discern research gaps within systematic reviews, thereby guiding subsequent investigative trajectories. ^23^ This study identified the most significant and prolific journals and authors, revealed the primary research organisations focused on MI, and monitored the advancement of knowledge regarding MI in the agricultural sector through bibliometric methodologies. ^24^ Using traditional bibliometric rules like Bradford’s Law, we found the journals that publish the most papers on international agricultural marketing. ^25^

The study followed the PRISMA 2020 (Preferred Reporting Items for Systematic Reviews and Meta-Analyses) guidelines to ensure a systematic and clear review process. PRISMA is one of the most popular frameworks for publishing systematic reviews and meta-analyses because it gives a structure that makes things clearer and easier to repeat. This guideline helped organise the steps of finding, screening, checking eligibility, and including articles. This made the findings more valid and less biassed. The research was conducted in accordance with the PRISMA 2020 declaration, which ensures precise, transparent, and uniform reporting of methodologies and results, in alignment with international standards of exemplary scientific practice. ^26^

### 2.1 Data

The bibliometric analysis was conducted using publications retrieved from Scopus, Dimensions and WoS. The selection of these databases was based on their comprehensive coverage and scientific rigour in applied social sciences, agricultural economics and management studies. This choice helped to ensure the representativeness and quality of the set of publications analysed. ^27, 28^

The inclusion criteria were as follows: original peer-reviewed articles published between 1960 and 2024 (with the time span adjusted to the availability of each database). The studies had to be framed within the fields of economics, business administration, international business or agricultural sciences, and address topics related to MI and the international marketing or export of agricultural products.

The exclusion criteria were: documents published outside the defined time range; publications belonging to subject areas unrelated to the scope of the study; documents without full-text access; and studies that did not address market intelligence, international marketing, export performance, or the commercialisation of agricultural products in international markets.

### 2.2 Procedure

**2.2.1 PRISMA 2020 process flow**

Following the PRISMA 2020 statement, the selection process was structured in four phases: identification, selection, eligibility, and inclusion. Table 1 shows the search process that was carried out using a combination of Boolean operators (see Table 1).

**Table 1.** Databases for systematic literature review.

Database	Search protocol	Documents	Search date
Scopus	TITLE-ABS-KEY(("market intelligence" OR "marketing intelligence" OR (market W/3 intelligence) OR ("competitive intelligence" W/3 market*)) AND (export* OR "export performance" OR exporting OR "export market*" OR "export* strateg*" OR "export* compet*"))	99	20 September 2025
Dimensions	("market intelligence" OR "marketing intelligence" OR "competitive intelligence" OR "market sensing" OR "marketing information system*") AND (export* OR exporting OR "export performance" OR "export strateg*" OR "export market*" OR "export competit*")	39,680	21 November 2025
WOS	TS=(("market intelligence" OR "marketing intelligence" OR "competitive intelligence" OR (market NEAR/2 intelligence) OR "market sensing" OR "marketing information system*") AND (export* OR exporting OR "export performance" OR "export strateg*" OR "export market*" OR "export competit*"))	188	20 September 2025
Total, documents		39,967	

*Note.* Data obtained from the Scopus, Dimensions, and WoS databases (2025).

In the identification phase, searches were conducted (Scopus: 99; Dimensions: 39,680; WoS: 188). In Scopus, the search strategy was ‘market intelligence’ OR ‘marketing intelligence’ OR ‘market W/3 intelligence’ OR ‘competitive intelligence’ W/3 market* AND export* OR ‘export performance’ OR exporting OR ‘export market*’ OR ‘export* strateg*’ OR ‘export* compet*’, which yielded 99 records. For Dimensions, the protocol ‘market intelligence’ OR ‘marketing intelligence’ OR ‘competitive intelligence’ OR ‘market sensing’ OR ‘marketing information system* AND’export* OR exporting’ OR ‘export performance’ OR ‘export strateg*’ OR ‘export market*’ OR ‘export competit" detected 39,680 documents. In Web of Science, the protocol ‘market intelligence’ OR ‘marketing intelligence’ OR ‘competitive intelligence’ OR ‘market NEAR/2 intelligence’ OR ‘market sensing’ OR ‘marketing information system’ AND ‘export* OR exporting OR’export performance‘OR’export strateg* OR ‘export market*’ OR ‘export competit’ identified 188 records. The sum of these results from the three platforms amounted to a total of 39,967 initial records, which were then subjected to a rigorous refinement and selection process, following the pre-established inclusion and exclusion criteria for the systematic review. Each database was then filtered by year (1960-2024) and document type (scientific articles only), excluding subject areas unrelated to MI and agro-exports. After this initial refinement, 1,465 items were obtained. These records were compiled, duplicates were removed, and unrecoverable documents were discarded, resulting in 1,454 unique documents. During the selection phase, titles and keywords related to MI, competitive intelligence, technology watch, and consumer knowledge were examined. This resulted in the discarding of 1,044 articles with information unrelated to the topic, leaving 393 articles. After a more thorough review of titles and abstracts during the eligibility phase, the number of articles was reduced to 112 that met the conceptual and methodological standards. After conducting a thematic and methodological assessment to reduce bias and ensure quality, a final corpus of 83 articles was created for bibliometric analysis. This process followed the PRISMA 2020 guideline to ensure that it was open, traceable, and scientifically sound. ^29^

**2.2.2 Assessment of risk of bias**

**Figure 1.**
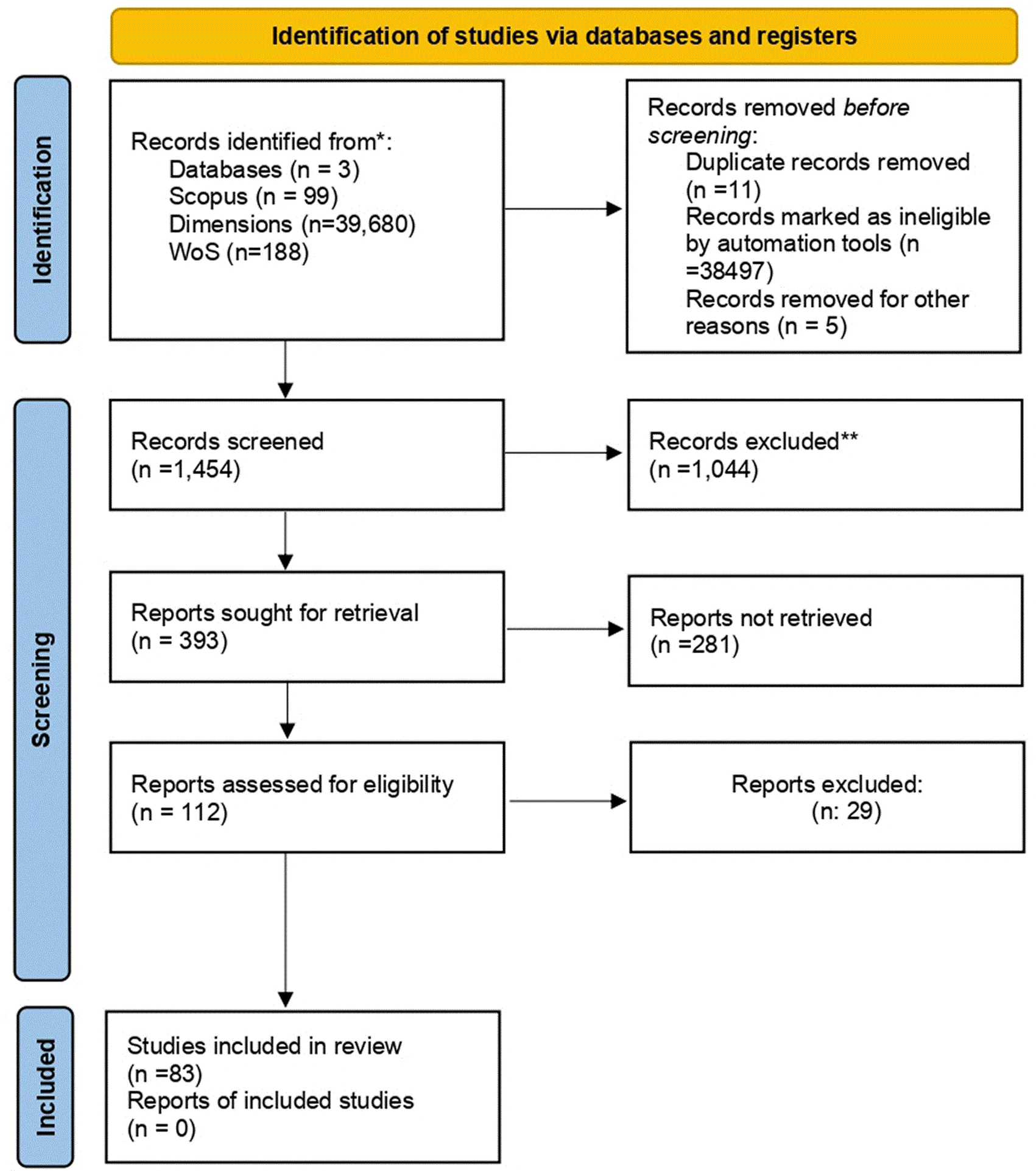
Prisma 2020 flow chart for selecting and including systematic review documents.

**Figure 2.**
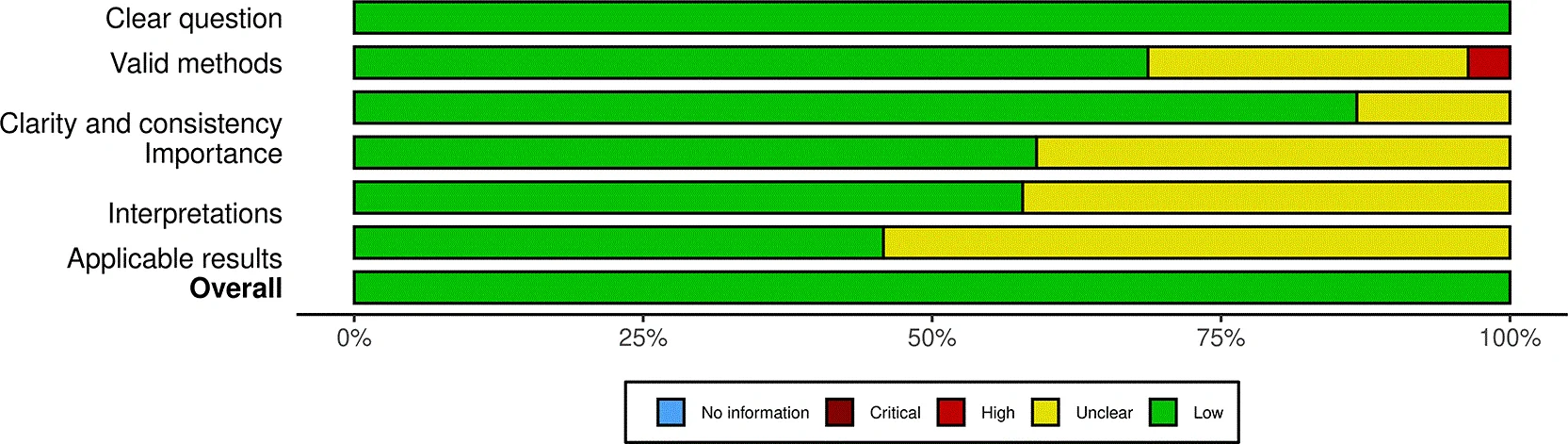
Weighted bar charts of the distribution of bias risk judgements within each bias domain.

In Figures 1 and 2, as part of the systematic review, the risk of bias was analysed to ensure that the studies included in the analysis were methodologically sound. An assessment matrix comprising theoretical, methodological, and empirical criteria was used to assess the risk of bias of the 83 publications included in the research. The aim of this review was to assess the consistency of the problem, methodology, and results of each study, along with the validity of data collection instruments, the quality of discourse, and the adequacy of conclusions. The clarity of the research question, the consistency and reliability of sources, the applicability of results to contemporary contexts, and their relevance were also evaluated. Using these criteria, a “traffic light” system was developed to classify risk as low, moderate or high. Studies classified as low risk were distinguished by the presence of a clearly defined research question, the use of appropriate methodologies, and the alignment between the study objectives and the results obtained. Those classified as moderate risk had incomplete or limited information regarding methodology or interpretation. Finally, studies that presented biases in their design, lacked validity in their results, or had interpretations that were not adequately justified were considered high risk. This process ensured the systematic review of the quality and reliability of the final corpus, as well as the transparency, traceability, and methodological rigour of the research (see Figures 1 and 2).

The initial screening, which yielded a manageable number of articles, was carried out by thoroughly reading titles, abstracts, and keywords to verify their suitability for the research approach. The organisation of the information collected was conducted exclusively through the utilisation of Microsoft Excel spreadsheets, employing the PRISMA 2020 diagram as a structural framework. The risk of bias was evaluated using a traffic light code (green: low; yellow: moderate; red: high), which permitted a standardised and expeditious evaluation of methodological quality and defined the strength of the conclusions. ^30^ Moreover, this structured approach enabled the visualisation of processes in Bibliometrix and VOSviewer, thereby demonstrating patterns, collaborations and citation trends in the context of international agricultural trade. ^31^ The quantitative analysis was conducted using Bibliometrix, a tool developed at the University of Naples Federico II, ^32^ while VOSviewer, a software developed at the Centre for Science and Technology Studies at Leiden University, was employed to generate bibliometric maps of thematic clusters and author-relationships. ^33^

The values of honesty, scientific integrity and good practices in R&D&I, as outlined in the Research Ethics Code of the, ^34^ were adhered to during the preparation of this research. The emphasis was placed on methodological rigour, objectivity, respect for the intellectual property of the authors (APA 7th edition standards) and adaptation to the principles of originality and integrity.

The risk of bias in the included studies was assessed using a risk matrix and the Robvis tool. This methodology is recognised by the scientific community as a means of evaluating the internal validity of studies. Rather than relying on checklists or numerical scores, it focuses on exploring specific domains of bias. ^35^

**Figure 3.**
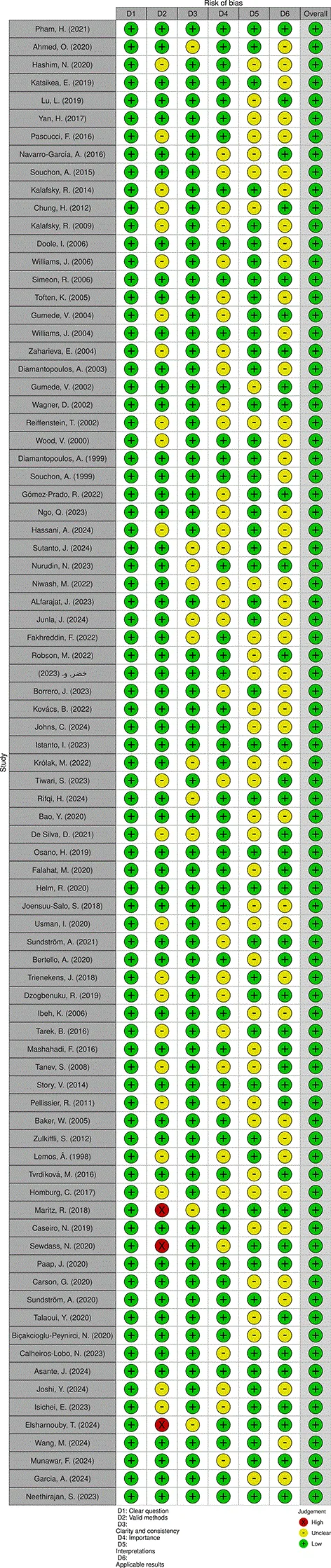
Traffic light charts of domain-level judgements for each individual result.

As illustrated in Figure 2 and Figure 3, the individual scores in six methodological domains are represented according to the traffic light system established in this review. This system utilises a colour-coding system to denote varying levels of risk, with green indicating low risk, yellow indicating moderate risk, and red indicating high risk. This system has been developed for the purpose of this study and is based on a quality matrix. In general, the majority of studies demonstrate a low risk of bias in the domains associated with the definition of the research objective (D1), methodological consistency (D2), and consistency between results and conclusions (D5), thus demonstrating adequate methodological rigour in the analysed corpus. However, moderate risks (yellow) were identified in certain studies within domains D3 (data quality and exhaustiveness) and D4 (validity of instruments). These findings indicated deficiencies in the methodological description and transparency of the analysis process in specific articles. A limited number of studies were found to present a high risk of bias, primarily due to inadequate methodological descriptions, the absence of instrument validity criteria, or the lack of clarity in the justification of results. Nevertheless, this does not compromise the robustness of the bibliometric and qualitative analysis of this study.

The study found that 76% of the studies had a low risk of bias, 19% had a moderate risk, and only 5% had a high risk. This conclusion suggests that the scientific evidence under examination is methodologically robust; however, it is essential to acknowledge that further research is necessary. The primary objectives of this enhancement should be the validation of empirical data and the standardisation of methodological reports.

The findings of this research substantiate the subsequent comparative synthesis across studies by reaffirming the intrinsic validity of the existing systematic review. This approach successfully circumvents potential biases that could otherwise compromise the theoretical and empirical synthesis of market intelligence within the context of international agricultural trade.

## 3. Results

**Figure 4.**
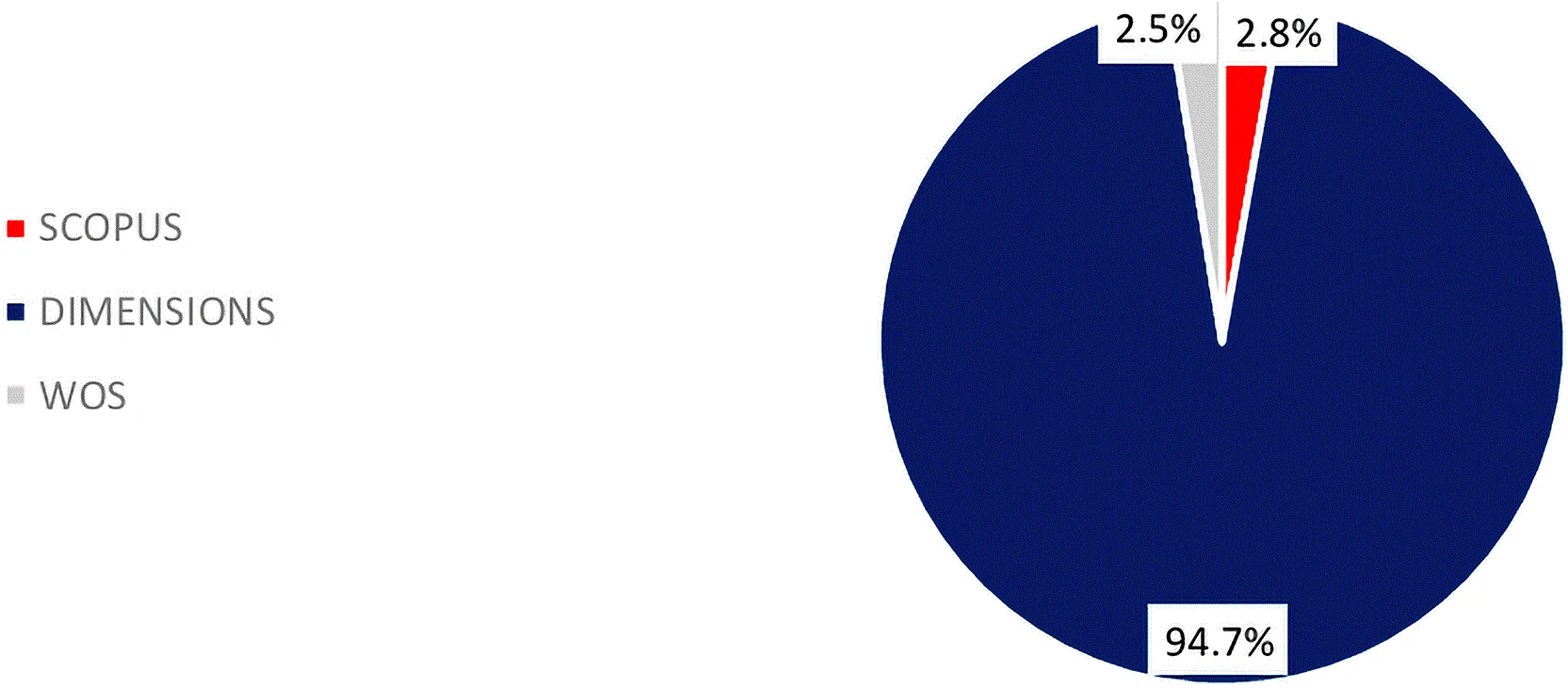
Percentage of total publications on market intelligence and marketing identified in Scopus, Dimensions, and WoS between 1960 and 2024.

Figure 4 shows the distribution of the 1,463 publications identified across the three databases during the 1960–2024 period. Dimensions accounted for the largest share, with 1,385 documents (94.7%), followed by Scopus with 41 publications (2.8%) and Web of Science with 37 (2.5%). This pronounced concentration reflects differences in database coverage and indexing breadth, particularly in retrieving literature related to market intelligence, marketing, and business performance. Accordingly, these percentages should be interpreted as the relative contribution of each database to the retrieved bibliographic set rather than as an indicator of the scientific quality or relevance of the publications (see Figure 4).

**Figure 5.**
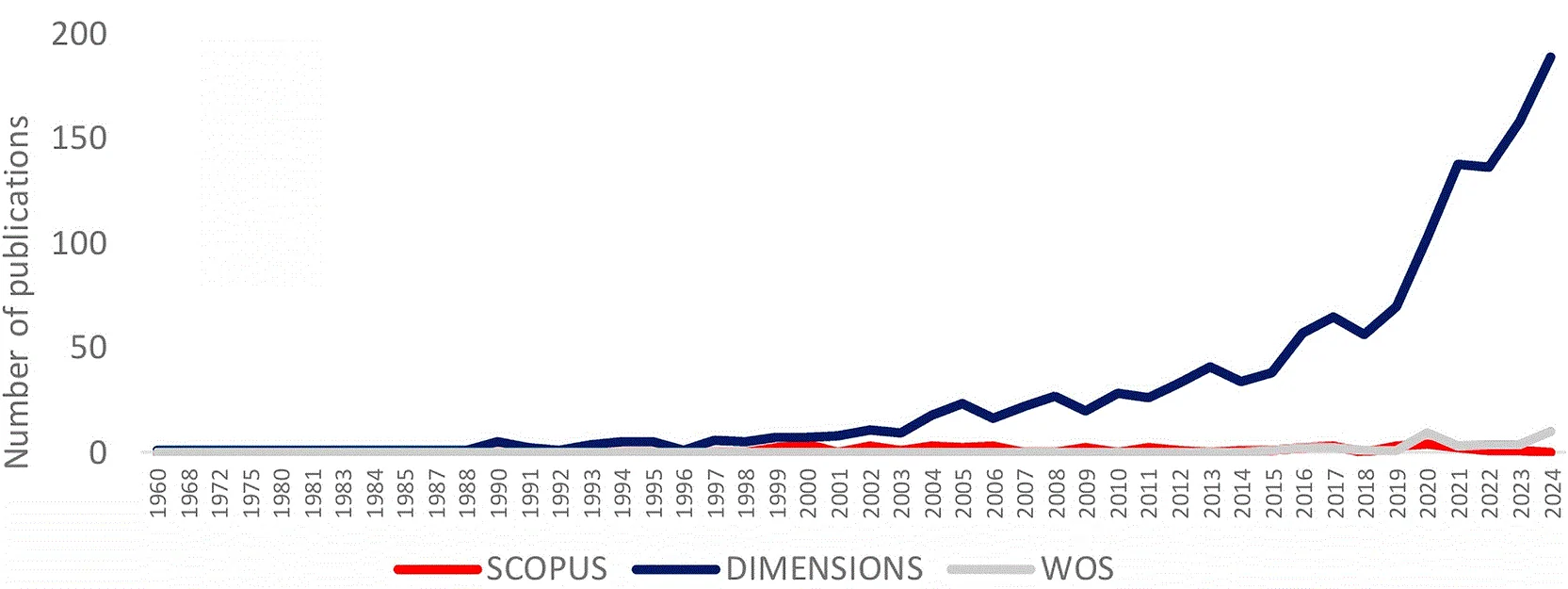
Trends in publications on the growth of market intelligence in marketing between 1960 and 2024.

Agro-export firms are increasingly optimising logistics and data analysis in order to improve their competitiveness in global markets, a trend supported by the growing availability of business intelligence tools. ^37^ As shown in Figure 5, there has been a marked increase in scientific production related to MI applied to marketing, particularly since 2018. This growth is closely associated with the acceleration of digital transformation in business processes, the expansion of data analytics and the adoption of new technologies in productive sectors. These developments have opened new lines of research focused on data-driven decision-making.

The predominance of publications indexed in Dimensions indicates that a significant proportion of recent contributions have been disseminated through open-access platforms, often with an interdisciplinary orientation. This suggests an expansion of the field towards practical applications in agricultural economics and international trade. The sustained increase in publications after the COVID-19 pandemic is also noteworthy, reflecting heightened concern in academic and business circles about how to mitigate uncertainty in volatile trading environments. In this context, MI has been strategically prioritised as a tool to strengthen export competitiveness (see Figure 5).

**Figure 6.**
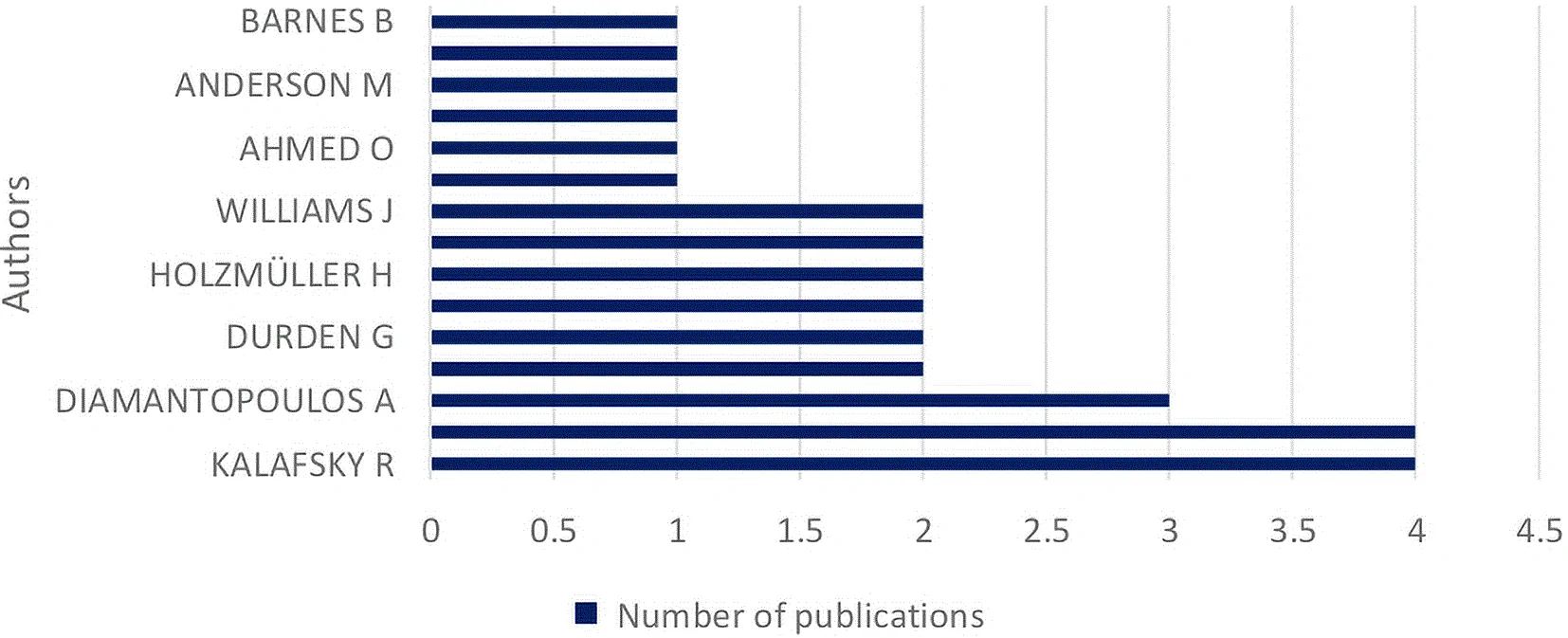
Main authors contributing to the collection, obtained from the Scopus database.

With regard to authorship, Figure 6 shows that scientific output in Scopus is relatively fragmented. Most authors have published fewer than five articles on MI in international marketing, indicating that a consolidated academic community has not yet formed around this topic. The most prominent authors, such as Kalafsky R. and Diamantopoulos A., come from fields such as international trade and territorial competitiveness. This suggests a dominant orientation towards economic geography and market studies, rather than towards technological or digital models of MI. Overall, the pattern points to an emerging field with dispersed academic leadership, which opens opportunities for future research particularly in Latin America linking theory and application in productive sectors such as agro-exports (see Figure 6).

**Figure 7.**
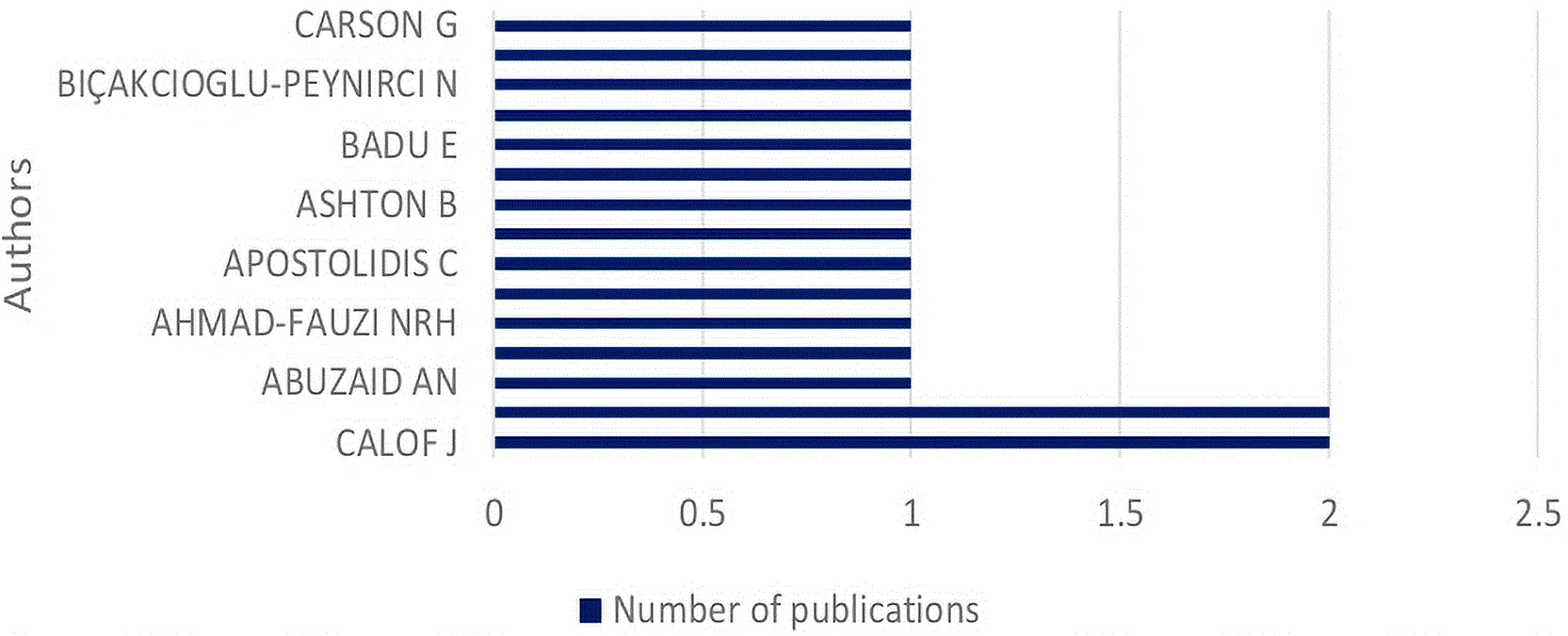
Main authors contributing to the collection, obtained from the DIMENSIONS database.

A survey of the Dimensions database reveals a paucity of scientific output on market intelligence in international trade, with the academic field remaining in its infancy. Indeed, no author has published more than two articles on the subject. The primary author, Calof J., has published extensively on the subjects of competitive intelligence and internationalisation strategies. His work indicates that Dimensions is more oriented towards strategic business analysis than applications in the agro-export sector. Furthermore, the geographical dispersion of authors indicates a high level of participation from Europe and the Middle East, contrasting with the predominance of Asian authors observed in Dimensions. This finding underscores the notion that scientific output exhibits variations in accordance with the orientation and scope of each database. In summary, the results demonstrate that Dimensions remains predominantly academic and theoretical, with a bias towards competitive intelligence studies as opposed to practical applications in agricultural chains for consumption (see Figure 7).

**Figure 8.**
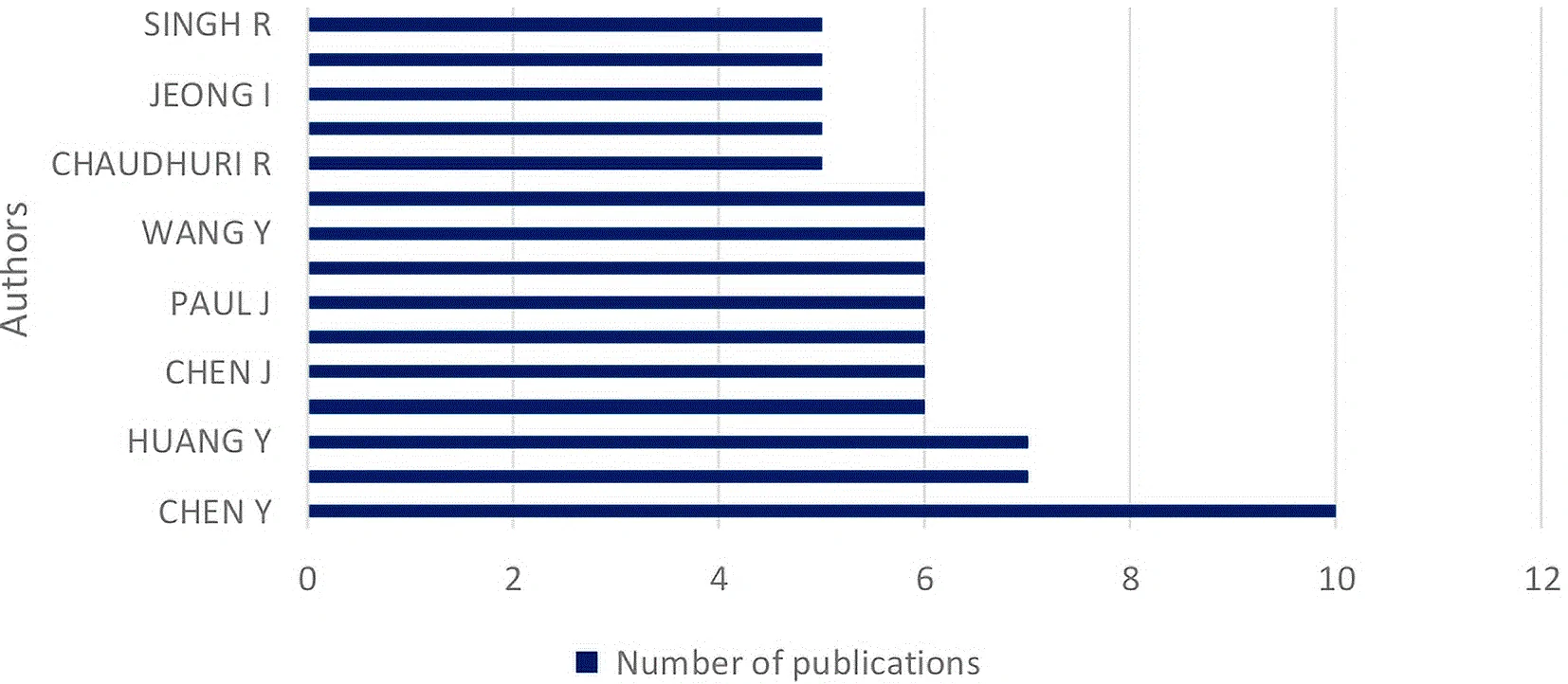
Main authors contributing to the collection, obtained from the Web of Science database.

In contrast, Figure 8 shows that academic output in Web of Science is more strongly concentrated among authors of Asian origin, especially from China and India. Within the group of authors analysed, Chen Y., Huang Y. and Chen J. stand out for their productivity in the area of MI for marketing. This pattern reflects a regional shift in intellectual leadership towards Asia, a region characterised by rapid growth in agro-exports and high investment in R&D. The leading authors in this database tend to adopt a pragmatic perspective, closely linking MI to business competitiveness and the digitalisation of agri-food chains. These trends suggest that Asia is consolidating its position as a key producer of knowledge on MI, surpassing Europe and North America in the volume and dynamism of research in this field ( Figure 8).

**Figure 9.**
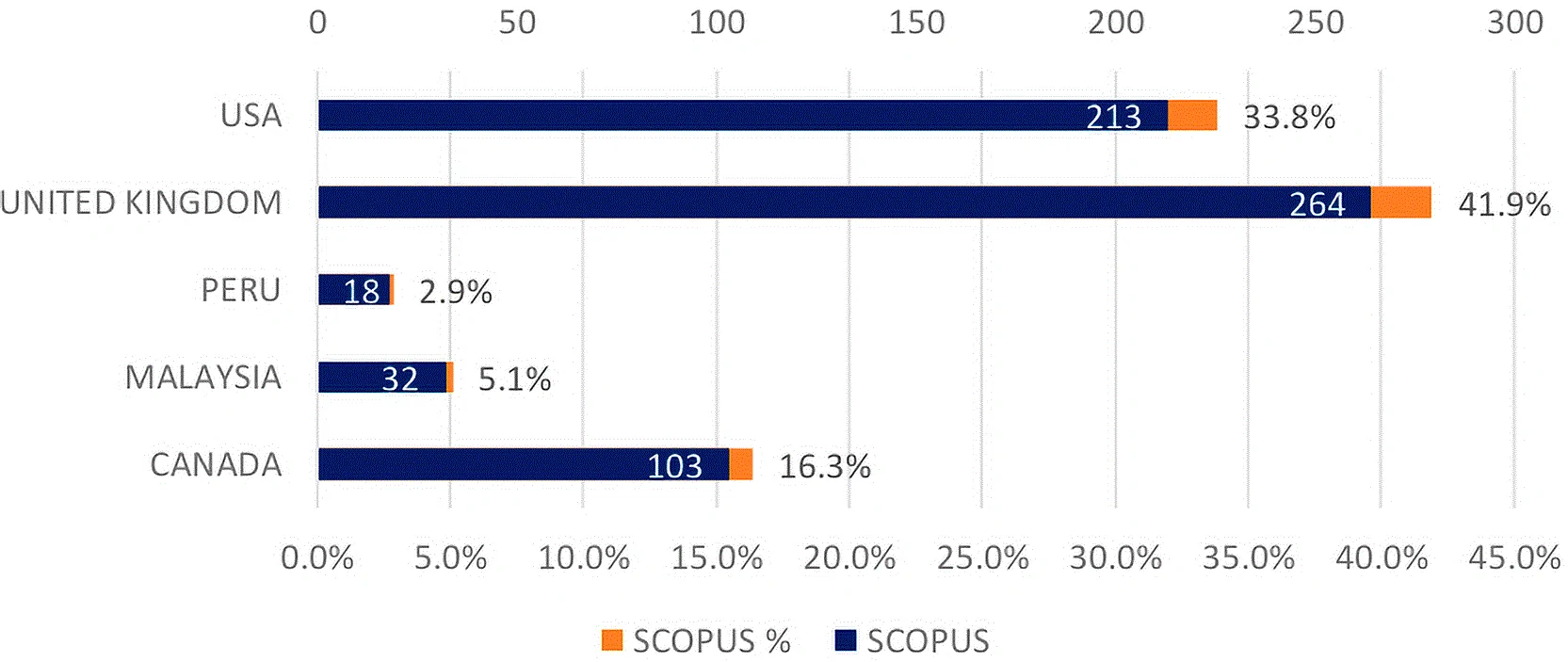
Publications by country on market intelligence in marketing, obtained from the Scopus database.

As demonstrated in Figure 9, the United Kingdom and the United States are the leading countries in terms of academic production in the field of market intelligence applied to marketing contexts within the Scopus database. This finding is indicative of the preeminence of countries with a long-standing tradition of research in business sciences and international trade. This leadership is predicated on the strength of their academic ecosystems, with high investment in applied R&D and extensive international collaboration networks in the area of strategic management and business competitiveness. Conversely, countries such as Canada and Malaysia demonstrate low levels of participation, while Peru and other Latin American countries exhibit minimal scientific output in this domain, despite their economies being heavily reliant on agro-exports. This scientific discrepancy indicates that there are knowledge gaps, and that market intelligence has not been fully developed from geographical perspectives, particularly in Latin America. This provides a foundation for future research to identify the utilisation of market intelligence in emerging economies and agricultural export sectors, where the use of information is crucial for competitiveness (see Figure 9).

**Figure 10.**
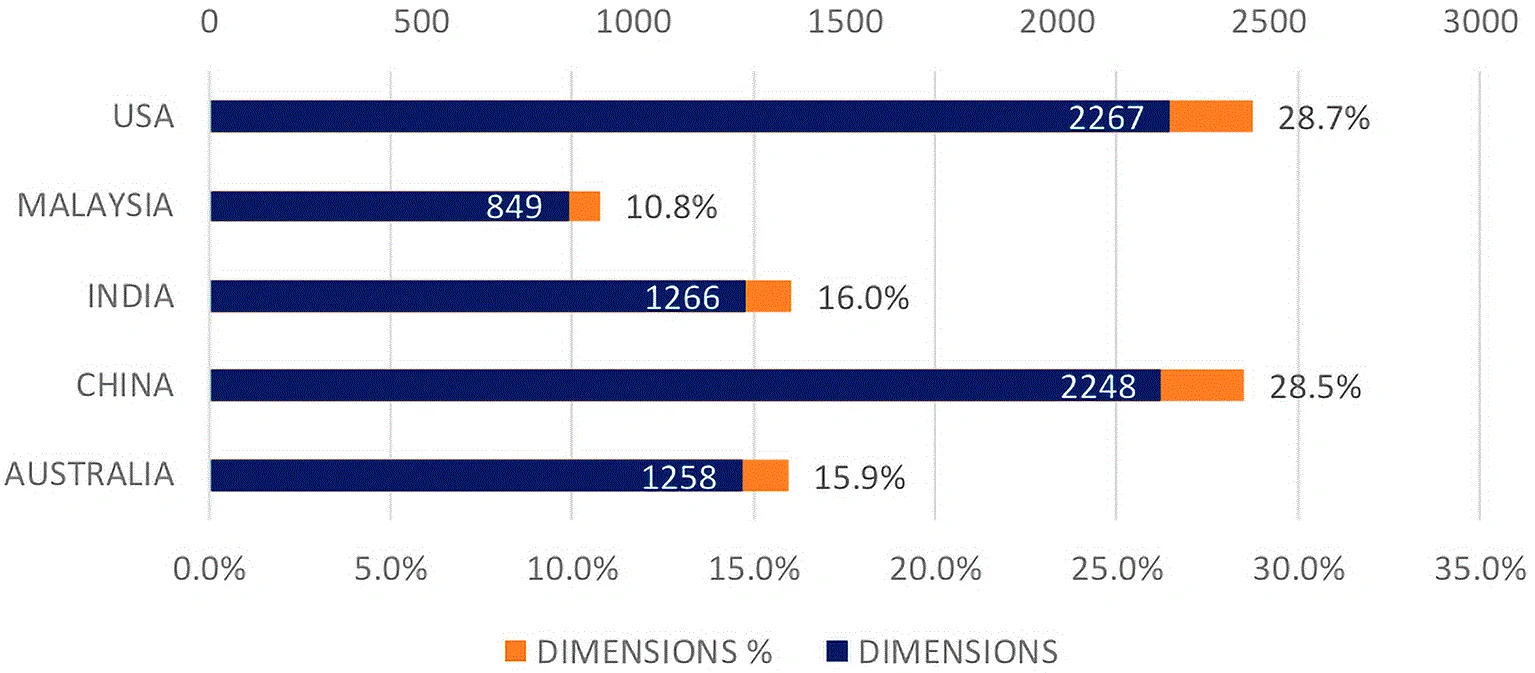
Publications by country on market intelligence in marketing, obtained from the Dimensions database.

Figure 10 shows that the United States and China are the two countries that do the most market intelligence research in marketing, according to the Dimensions database. India, Australia, and Malaysia are not far behind. This pattern shows that economies with a strong focus on technology and a growing role in global supply chains are driving progress in this field. This is in line with how data analytics and digitisation are improving market intelligence in these areas. The rise of China and India as major economic powers has changed the focus of academic leadership to Asia, where market intelligence is being used as a way to make exports more competitive in fields like agriculture, manufacturing, and technology. Scopus shows Europe as a leader in scientific productivity, but Dimensions focusses more on scientific activity in developing countries. This suggests that the field is moving towards a more practical and market-oriented approach. On the other hand, the fact that Latin American countries are not well represented in this database shows that there is a lack of academic resources in the region that makes it harder to create knowledge that is useful for agro-exporting economies like Peru, Colombia, and Mexico. This situation highlights the need to promote market intelligence research from local viewpoints, especially in agricultural sectors facing international challenges in competitiveness and innovation (see Figure 10).

**Figure 11.**
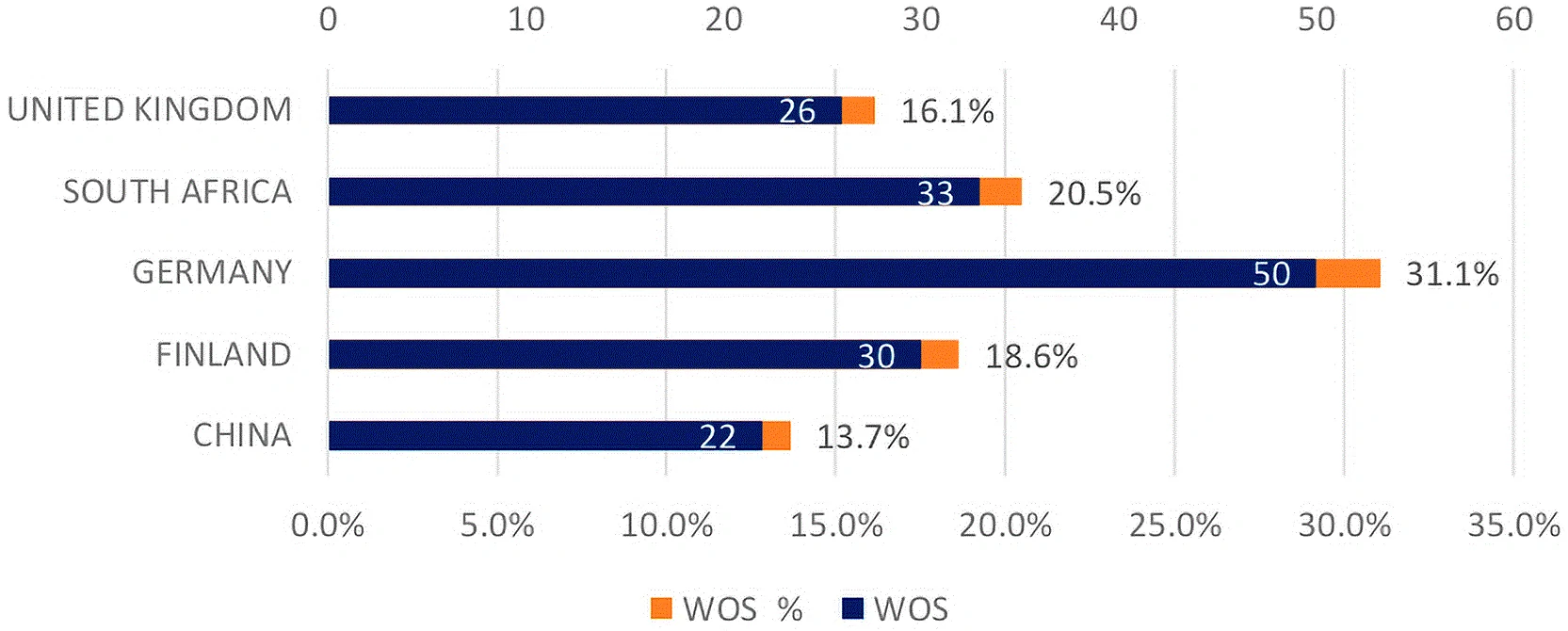
Publications by country on market intelligence in marketing, obtained from the Web of Science database.

Figure 11 shows that Germany and South Africa are at the top of the list for scientific output in marketing-oriented market intelligence indexed in Web of Science. The United Kingdom and Finland follow them. There is evidence that China’s participation is going down, which is different from what Dimensions says. This finding indicates a more theoretical and strategic focus in WoS, linked to business management, competitiveness, and organisational studies, which are predominant in Europe. South Africa’s participation is significant, as its prominent presence illustrates the interest of emerging economies in utilising market intelligence to improve their engagement in international markets and to support unconventional export sectors. However, the minimal contribution of Latin American countries highlights a significant academic deficiency in the region, which obstructs the progress of knowledge relevant to their agro-export environments. Unlike the Scopus and Dimensions databases, which show that most of the publications come from the US and China, the results from the WoS database show that it favours publications that show a lot of academic rigour. This observation seems to support the idea that countries with strong research institutions and stable networks of collaboration are more likely to show up in WoS results (see Figure 11).

Bradford’s Law, applied to this corpus of publications, would allow us to identify the most productive journal clusters and the areas of lowest output, thus revealing the concentric structure of information. ^38^

As illustrated in Table 2, the principal scientific domains of market intelligence within the Scopus database are identified in accordance with Bradford’s Law. Zone 1 is home to a number of highly esteemed journals specialising in strategic marketing and business competitiveness, including the International Marketing Review, Marketing Intelligence and Planning, and the European Journal of Marketing. This finding indicates that the generation of knowledge in the field of market intelligence is predominantly derived from the domain of business marketing, rather than from agricultural economics or agricultural sciences. This observation serves to reinforce the business and competitive nature of market intelligence. Zone 2, meanwhile, brings together journals that, although less prolific, adopt interdisciplinary approaches by combining issues of entrepreneurship, organisational management, innovation and sustainability. This suggests the necessity of extending the field to new areas of application. This dispersion indicates a shift in market intelligence from a 100% commercial vision to a more strategic and cross-cutting one, closely linked to decision-making in globalised environments (see Table 2).

**Table 2.** Bradford law with information obtained from the Scopus database in Bibliometrix.

Magazine	Ranking	Frequency	Cumulative frequency	Zone
International marketing review	1	6	6	Zone 1
Marketing intelligence and planning	2	4	10	Zone 1
European journal of marketing	3	2	12	Zone 1
Geojournal	4	2	14	Zone 1
Journal of small business and enterprise development	5	2	16	Zone 2
Sustainability (switzerland)	6	2	18	Zone 2
Asia pacific business review	7	1	19	Zone 2
Economic geography	8	1	20	Zone 2
Industrial marketing management	9	1	21	Zone 2
International business review	10	1	22	Zone 2
International economics and economic policy	11	1	23	Zone 2
International journal of contemporary hospitality management	12	1	24	Zone 2
International journal of entrepreneurship	13	1	25	Zone 2
International small business journal	14	1	26	Zone 2
Journal for global business advancement	15	1	27	Zone 2

*Note.* Data obtained from Bibliometrix (2025).

As illustrated in Table 3, which has been constructed in accordance with Bradford’s Law for Dimensions, Zone 1 comprises a selection of interdisciplinary management and strategy journals, including Sustainability, Journal of Business & Industrial Marketing, Industrial Marketing Management, European Journal of Innovation Management, and Journal of Business Research, amongst others. This development indicates a shift in the focus of market and marketing research towards sustainability, innovation, and competitive strategy, extending beyond the conventional marketing paradigm. It is evident from the publications of journals such as Technological Forecasting and Social Change and International Journal of Production Economics that the field is associated with technological change and the supply chain, in a manner analogous to the growth observed in data analysis and business digitalisation since 2018. The incorporation of open-access journals, such as Sustainability, underscores the extensive dissemination of content, a hallmark of Dimensions. The distribution by zone indicates an expanding and practical field, with a focus on applied results. However, the absence of specialised agricultural journals in Zone 1 suggests potential opportunities for the application of market intelligence in the agro-export sector, particularly in emerging economies (see Table 3).

**Table 3.** Bradford law with information obtained from the Dimensions database in Bibliometrix.

Magazine	Ranking	Frequency	Cumulative frequency	Zone
Sustainability	1	40	40	Zone 1
Journal of business and industrial marketing	2	36	76	Zone 1
Industrial marketing management	3	33	109	Zone 1
European journal of innovation management	4	28	137	Zone 1
Journal of business research	5	24	161	Zone 1
Cogent business & management	6	19	180	Zone 1
Business strategy and the environment	7	18	198	Zone 1
Journal of small business and enterprise development	8	17	215	Zone 1
Technological forecasting and social change	9	17	232	Zone 1
European journal of marketing	10	16	248	Zone 1
International marketing review	11	16	264	Zone 1
International journal of entrepreneurial behaviour & research	12	15	279	Zone 1
Journal of product innovation management	13	15	294	Zone 1
International journal of production economics	14	14	308	Zone 1
Benchmarking an international journal	15	13	321	Zone 1

*Note.* Data obtained from Bibliometrix (2025).

As demonstrated in Table 4, the application of Bradford’s Law to Web of Science reveals that Zone 1 encompasses journals specialising in strategy, innovation, and emerging markets, including Foresight and STI Governance, International Journal of Emerging Markets, International Marketing Review, and Technology Innovation Management Review. In contrast to the focus of Scopus and Dimensions on applied marketing, WoS places greater emphasis on development policies, competitiveness, and innovation management. This finding suggests that WoS adopts a more theoretical and strategic approach to market intelligence. The journals Competitiveness Review and Baltic Journal of Management illustrate the link between market intelligence in WoS and competitive economics and organisational development. This finding serves to reinforce the observation that the WoS database primarily encompasses macro and meso market studies, in contrast to the operational business analysis that characterises the Scopus and Dimensions databases. The paucity of specialised agricultural periodicals further exacerbates the disconnection between market intelligence and its practical application in the context of agro-export trade, particularly in developing countries. This research underscores the necessity to analyse this relationship from a sectoral and applied perspective (see Table 4).

**Table 4.** Bradford law with information obtained from the Wos database in Bibliometrix.

Magazine	Ranking	Frequency	Cumulative frequency	Zone
Foresight and sti governance	1	5	5	Zone 1
International journal of emerging markets	2	2	7	Zone 1
International marketing review	3	2	9	Zone 1
Technology innovation management review	4	2	11	Zone 1
Baltic journal of management	5	1	12	Zone 1
Competitiveness review	6	1	13	Zone 1
Construction economics and building	7	1	14	Zone 2
Creativity and innovation management	8	1	15	Zone 2
Eskisehir osmangazi universitesi iibf dergisi-eskisehir osmangazi university journal of economics and administrative sciences	9	1	16	Zone 2
Foundations of management	10	1	17	Zone 2
Heliyon	11	1	18	Zone 2
Humanities & social sciences communications	12	1	19	Zone 2
International journal of advanced and applied sciences	13	1	20	Zone 2
International journal of e-business research	14	1	21	Zone 2
International journal of research in marketing	15	1	22	Zone 2

*Note.* Data obtained from Bibliometrix (2025).

In order to comprehend Lotka’s law, it is imperative to undertake a thorough analysis of the productivity of authors specialising in market intelligence within the global trade of agricultural commodities. This methodology enables the identification of the most influential authors and the description of their publication patterns. ^39^ As demonstrated in Table 5, an analysis of author productivity in relation to Lotka’s Law reveals a significant concentration. Specifically, it is observed that 90% of authors in Scopus and Dimensions contribute a single market intelligence article in the domain of marketing, thereby significantly reducing the number of authors with two or more publications.

**Table 5.** Lotka’s law, developed from the Scopus, Dimensions and Web of Science databases in Bibliometrix.

SCOPUS			DIMENSIONS		WOS	
N.° Articles	N.° Authors	Frequency	N.° Authors	Frequency	N.° Authors	Frequency
1	83	90.2%	3163	90.0%	95	98%
2	6	6.5%	251	7.1%	2	2.1%
3	1	1.1%	66	1.9%	0	0
4	2	2.2%	18	0.5%	0	0
5	0	0	8	0.2%	0	0
6	0	0	7	0.2%	0	0
7	0	0	2	0.1%	0	0
10	0	0	1	0.03%	0	0

*Note.* Data obtained from Bibliometrix (2025).

This ‘long tail’ phenomenon is indicative of fields in the process of consolidation, suggesting an absence of a stable core of specialists who generate knowledge on a recurring basis. Instead, there is a predominance of sporadic and heterogeneous contributions from various disciplines. A greater proportion of ‘single-contribution authors’ has been demonstrated to imply fragmented academic leadership, a paucity of continuous research agendas, and a dependence on one-off collaborations to the detriment of established groups. In the Web of Science database, this concentration is even more pronounced, thereby accentuating the perception of a more selective and theoretical approach, with less recurrence of the same researchers (see Table 5).

The utilisation of VOSviewer facilitates the generation of bibliometric maps, thereby elucidating the thematic interconnections and keyword clustering within the extant literature. ^40^ The findings indicated a correlation between the subjects of interest, including innovation, market orientation, export performance/yield, competitive advantage, and market intelligence/marketing information. These terms were also found to be significantly associated with three databases (Scopus, WoS, and Dimensions).

**Figure 12.**
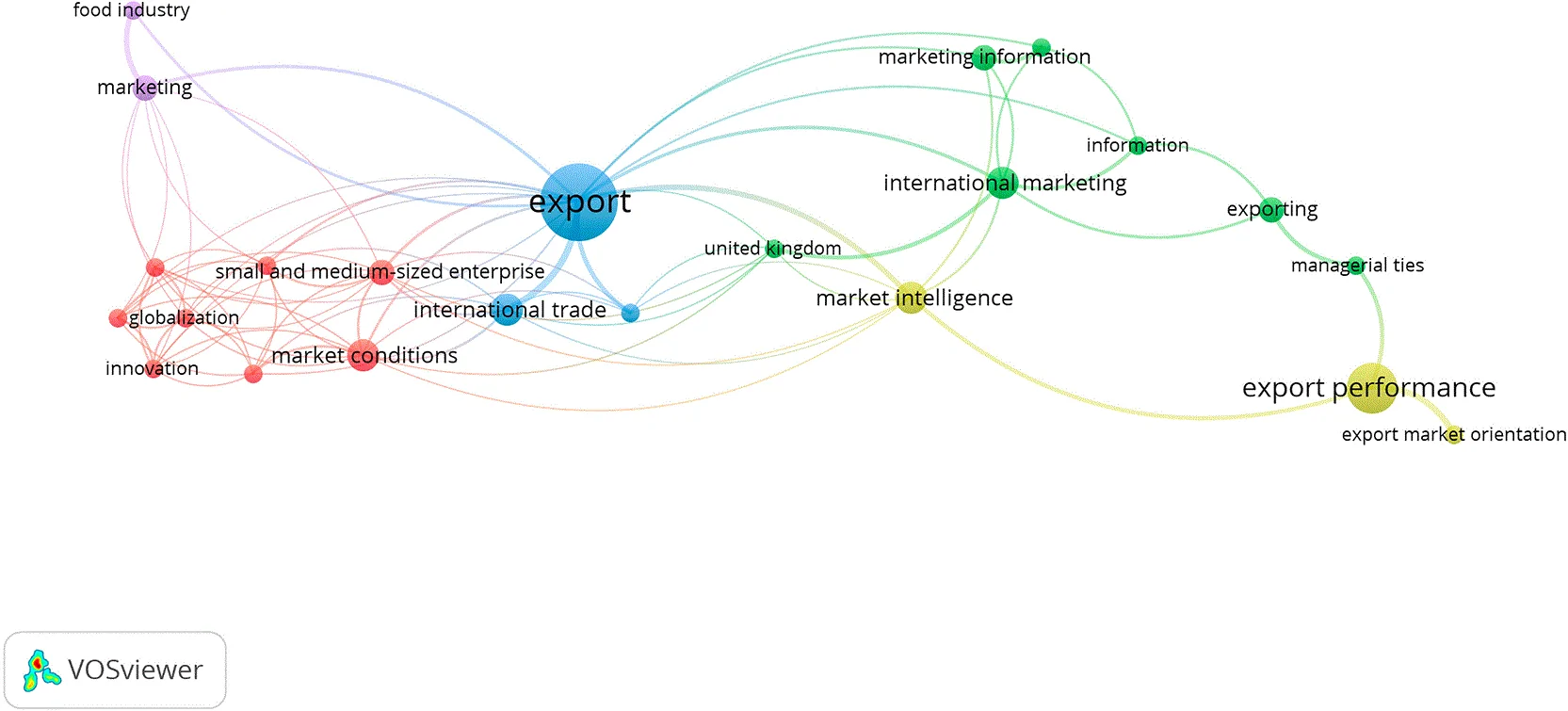
Semantic map of the relationship between market intelligence and marketing, obtained from the open-source programme VOSviewer, with metadata from Scopus.

As illustrated by Figure 12, a VOSviewer semantic map demonstrates the conceptual structure of the field of study, created from Scopus metadata. The central term is “export”, thus confirming the focus on export performance as a result of market intelligence. The population can be divided into three main clusters: The green cluster groups the concepts of “market intelligence”, “marketing information” and “international marketing”, thus suggesting that market intelligence is a strategic tool for business decisions. The red cluster associates “innovation”, “market conditions”, “SMEs” and “international trade”, thereby establishing a linkage between market intelligence and competitiveness, innovation in SMEs and adaptation to global markets. Finally, the yellow cluster synthesises the concepts of “export performance” and “market orientation”, reflecting the interest in the impact of market intelligence on results. In summary, the extant literature in this field has addressed a causal line: market intelligence → strategic decisions → export results, emphasising market information as a global competitive advantage (see Figure 12).

**Figure 13.**
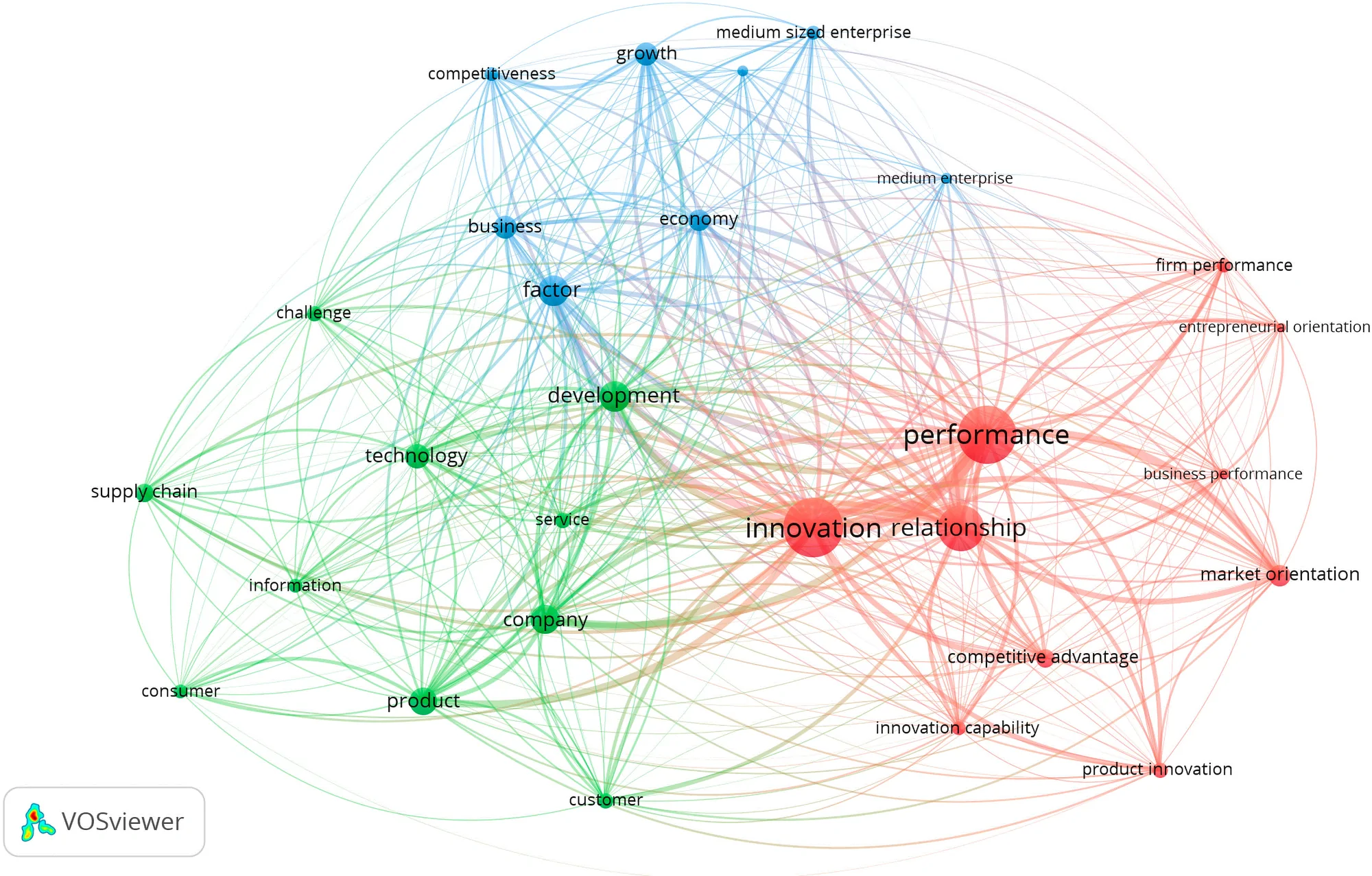
Semantic map of the relationship between market intelligence and marketing, obtained from the open-source programme VOSviewer, with metadata from Dimensions.

As illustrated in Figure 13, which was created using metadata from Dimensions, the conceptual structure of the field is represented. The terms “performance” and “innovation” are frequently cited, indicating a correlation between market intelligence and organisational enhancement, competitiveness, business outcomes and strategic decision-making. Three distinct groups can be identified: the red cluster, which refers to export capacity, competitive advantage and market orientation, verifying the influence of market intelligence on export performance. The green cluster is characterised by the integration of technological capacity, knowledge management and the innovation system, with market intelligence being integrated with knowledge management and innovation supported by digital technologies. The blue cluster encompasses the subjects of SMEs, competitiveness, business model and internationalisation; as such, a significant proportion of research focuses on the exporting activities of SMEs that wish to compete. The map illustrates an evolution towards explanatory models of competitiveness, in which market intelligence is pivotal to innovation, internationalisation and competitive advantage (see Figure 13).

**Figure 14.**
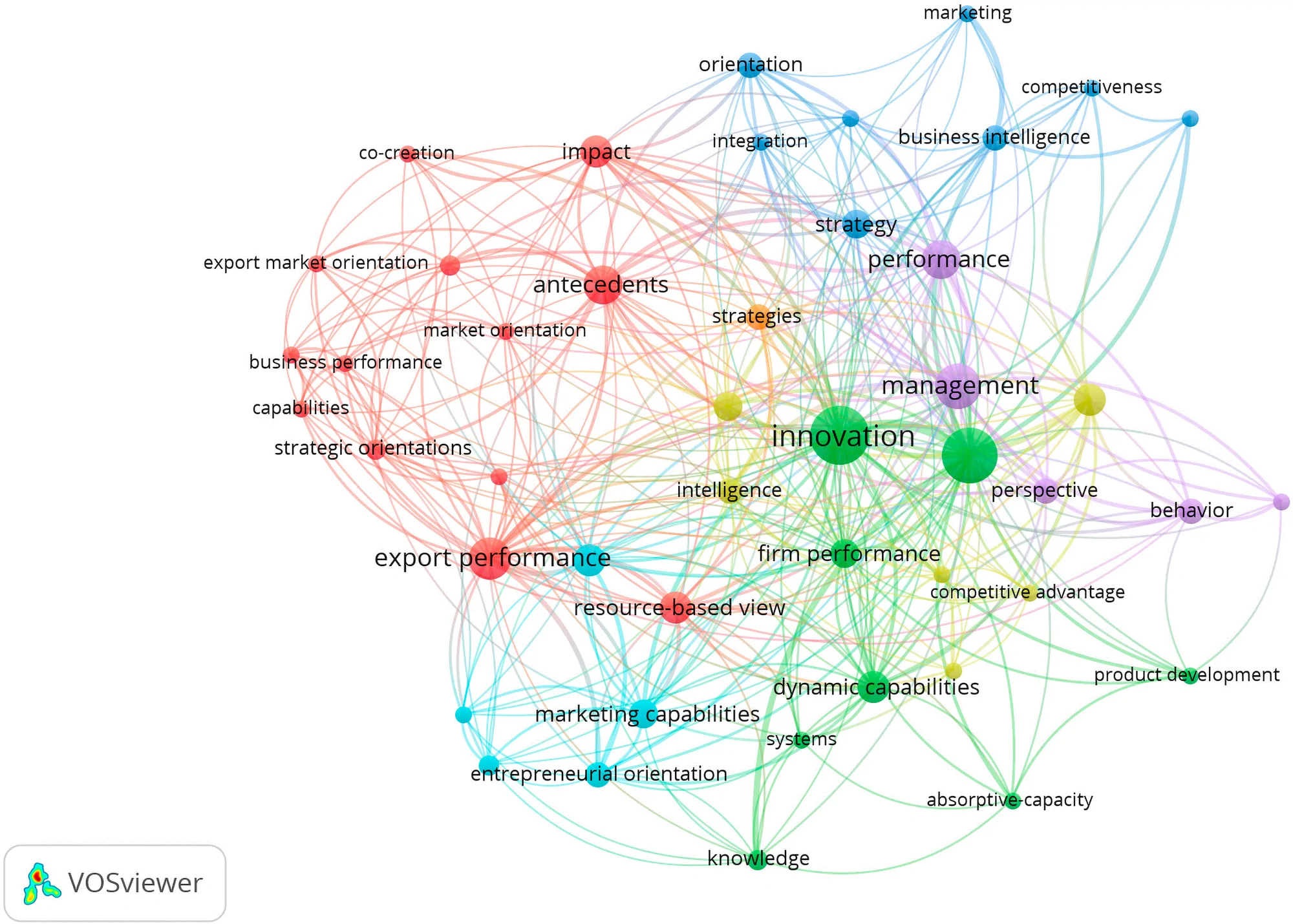
Semantic map of the relationship between market intelligence and marketing, obtained from the open-source programme VOSviewer, with metadata from WOS.

As demonstrated in Figure 14, the semantic map of Web of Science is significantly more extensive than those of Scopus and Dimensions. The terms ‘innovation’, ‘business performance’ and ‘management’ are recurrent, which suggests that WoS approaches market intelligence from high-level theoretical frameworks, such as competitive advantage, strategic management and resource and capability theory. The analysis identifies four primary groups: the green ones refer to RBV, AC and KM, indicating that WoS has already incorporated market intelligence into the strategic models of organisational competitiveness. The red block encompasses the following: ‘export performance’, ‘antecedents’, ‘market orientation’ and ‘business performance’. This serves to confirm that the optimisation of performance in international markets is one of the primary research lines. The blue block incorporates terms such as “competitiveness”, “strategy” and “company”, reflecting the manner in which the subject is addressed in globalised and constantly changing environments. The yellow team combines the concepts of “systems”, “perspective” and “product development”, sharing authentic case studies on product management and business development. In contrast to the Scopus and Dimensions semantic maps, the WoS semantic map is more aligned with theoretical concepts and makes greater use of explicit conceptual models, indicating a more theoretical and less descriptive approach (see Figure 14).

The collaborative interpretation of the clusters suggests that they do not represent discrete streams of study, but interconnected components of a wider strategic process. The informational background enabling enterprises to identify changes in demand, market conditions and worldwide opportunities is built on concepts of market intelligence, marketing information and knowledge management. This ability is associated with clusters of innovation, dynamic capacities, absorptive capacity and market orientation, which are the organizational processes thru which information is absorbed and converted into business decisions. These capabilities, in turn, converge with clusters associated with internationalization, competitiveness, business performance and export performance, revealing a conceptual sequence in which market intelligence facilitates knowledge acquisition and processing, strengthens strategic adaptation and supports decisions oriented toward outcomes in international markets. Within the framework of agro-exportation, this interconnectivity can be translated into concrete decisions in the identification of target markets, adaptation of supply to international demand, monitoring of competitors and prices, customer segmentation, management of channels and adjustment of internationalization strategies. The maps jointly imply that the contribution of market intelligence to export success does not work in an isolated mechanism, but thru its interplay with organizational competencies, innovation and strategic market orientation.

A comparative analysis of the databases reveals discrepancies in their trajectory, focus, and maturity. Dimensions has demonstrated a marked increase in the number of publications in recent times, a development that has been propelled by the application of research findings and the adoption of an open access policy. Scopus maintains moderate production, while Web of Science is more selective. Geographically, the United States, the United Kingdom and China are the leading countries in terms of scientific production, but new centres are emerging in India, Malaysia and South Africa, especially in Dimensions and WoS. The Latin American share is comparatively minor, a discrepancy given the significance of agricultural exports in the region. In the world of academic journals, Scopus is more interested in strategic marketing, while Dimensions is more interested in innovation and applied management. WoS, on the other hand, is mostly about global theoretical and strategic approaches. This highlights the inherently interdisciplinary nature of market intelligence research, which has transitioned from a primarily descriptive methodology to one increasingly defined by strategic models associated with innovation and competitiveness. Lotka’s Law shows that there is a lot of variation and not much academic consolidation. Most authors only publish one article, which shows that leadership is spread out and there are no clear research strands. Finally, semantic maps reveal a thematic convergence in market intelligence as a basis for strategic information, decision-making for export performance, and innovation as a source of competitive advantage. However, there is a paucity of specific agricultural or agro-export terminology, which suggests that the issue has not been addressed sectorally, especially in developing countries.

A comprehensive analysis of the extant scientific literature has identified three areas of knowledge that have contributed significantly to the development of market intelligence in the field of agricultural trade. These conceptual axes are fundamental to expanding existing knowledge and laying the foundations for future research in this field. In this sense, market intelligence is recognised as an indispensable factor for the future of logistics, as illustrated in Table 6.

**Table 6.** Results of the individual studies.

Authors	Contribution block	Market intelligence mechanisms for marketing
Souchon et al. (2015) ^41^; Williams (2006) ^42^; Williams & Chaston. (2004) ^43^; Diamantopoulos et al. (2003) ^44^; Reiffenstein et al. (2002) ^45^; Wood & Robertson (2000) ^46^; Diamantopoulos & Souchon (1999) ^47^; Mahmoud & Mahmoud (2023), ^48^ Souchon & Diamantopoulos (1999) ^49^; Pellissier & Kruger (2011) ^50^; Tvrdíková (2016) ^51^; Maritz & du Toit (2018) ^52^; Sewdass & Calof (2020) ^53^; Carson et al. (2020) ^54^; Talaoui et al. (2020) ^55^; Calheiros et al (2023) ^56^; Asante et al. (2024). ^57^	Within the domain of agricultural exports, market intelligence is delineated as a systematic process of collecting, analysing and strategically interpreting relevant information for the purpose of informing commercial decisions in international markets. This methodology enables agricultural companies to discern the complexities of the global market, identify windows of opportunity for export and anticipate fluctuations in demand. In the contemporary business environment, it is widely acknowledged as a pivotal instrument for augmenting competitiveness, facilitating adaptation to international regulations, and promoting more effective data management.	The following mechanisms are of key importance: firstly, continuous monitoring of international prices and dynamics; secondly, a detailed examination of competition and distribution channels; thirdly, supervision of phytosanitary and regulatory requirements; and fourthly, incorporation of data on consumers and target markets. These strategic procedures are imperative for effective decision-making, thereby minimising uncertainty and optimising the marketing of agricultural products in a global context.
Ahmed & Sallam. (2020) ^58^; Lu et al. (2019) ^59^; Kalafsky & Gress (2014) ^60^; Kalafsky (2009) ^61^; Simeon (2006) ^62^; Gumede (2004) ^63^; Zaharieva et al. (2004) ^64^; Gumede & Rasmussen (2002) ^65^; Hassani & Blais (2024) ^66^; Sutanto et al., (2024) ^67^; Nurudin (2023) ^68^; Junla & Naipinit (2024) ^69^; Borrero (2023) ^70^; Johns et al. (2024) ^71^; Tiwari et al. (2023) ^72^; De Silva et al. (2021) ^73^; Osano (2019) ^74^; Sundström et al. (2021) ^75^; Trienekens et al. (2018), ^76^ Ibeh et al. (2006) ^77^; Mashahadi et al. (2016) ^78^; Lemos & Porto (1998), ^79^ Paap (2020), ^80^ Sundström et al. (2020), ^81^ Elsharnouby et al. (2024), ^82^ Wang et al. (2024), ^83^ Munawar et al. (2024), ^84^ González García et al. (2023) ^85^; Neethirajan (2023). ^86^	As demonstrated in the literature specialising in this field, the generation of intelligence pertaining to the market is of pivotal significance in the context of formulating efficacious strategies and tactics for the marketing of agricultural products. The implementation of this strategy will facilitate the adjustment of supply to international demand, the enhancement of logistics processes, and the consolidation of the competitive position of agro-export products. Furthermore, it facilitates decision-making in areas such as pricing, differentiation and segmentation, which contributes to the establishment of lasting and profitable commercial relationships.	The most relevant procedures include research into consumer preferences, global market segmentation, monitoring of prices and demand through digital platforms, and comparative analysis of competition and distribution channels. The implementation of these practices enables the optimisation of promotion, negotiation and logistics strategies, which translates into improved efficiency and profitability for agricultural marketing in international markets.
Pham & Petersen (2021) ^87^; Hashim et al. (2020) ^88^; Katsikea et al. (2019) ^89^; Yan et al. (2017) ^90^; Pascucci et al. (2016) ^91^; Navarro et al. (2016) ^92^; Chung (2012), ^93^ Doole et al. (2006) ^94^; Toften (2005) ^95^; Gómez-Prado et al. (2022), ^96^ Ngo (2023) ^97^; Niwash et al. (2022) ^98^; Alfarajat (2023) ^99^; Fakhreddin & Foroudi (2022) ^100^; Robson et al. (2023) ^101^; Kovács & Szakály (2022) ^102^; Rifqi et al. (2024) ^103^; Bao (2020), ^104^ Falahat et al. (2020) ^105^; Helm et al. (2020) ^106^; Joensuu et al. (2018) ^107^; Usman et al. (2020) ^108^; Bertello et al. (2021) ^109^; Dzogbenuku & Keel-son (2019) ^110^; Tarek et al. (2016) ^111^; Tanev & Bailetti (2008) ^112^; Story et al. (2015) ^113^; Baker & Sinkula (2005) ^114^; Zulkiffli & Perera (2012) ^115^; Homburg et al. (2017) ^116^; Caseiro & Coelho (2019) ^117^; Bicakcioglu & Ipek (2020) ^118^; Joshi et al. (2024) ^119^; Isichei et al. (2023). ^120^	Empirical evidence confirms that market intelligence has a positive and significant impact on the export performance of agricultural companies. It empowers companies to transform data into strategic decisions, respond more quickly to global markets, strengthen their customer focus and increase their profitability. Studies agree that building business relationships, managing knowledge and being responsive are factors that enhance the effectiveness of market intelligence in agro-exports.	The most notable mechanisms include the continuous evaluation of export performance and the analysis of profitability and market penetration indicators. Other mechanisms include the measurement of international customer satisfaction levels and the systematic review of strategies based on informative feedback. These mechanisms validate the effectiveness of market intelligence actions and promote the continuous improvement of agricultural product marketing.

*Note.* Prepared internally based on the analysis of scientific studies (2025).

Block 1: «Market Intelligence in Marketing: Fundamentals and Current Outlook», by authors Souchon et al. (2015) ^41^; Williams (2006) ^42^; Williams & Chaston. (2004) ^43^; Diamantopoulos et al. (2003) ^44^; Reiffenstein et al. (2002) ^45^; Wood & Robertson (2000) ^46^; Diamantopoulos & Souchon (1999) ^47^; Mahmoud & Mahmoud (2023) ^48^; Souchon & Diamantopoulos (1999) ^49^; Pellissier & Kruger (2011) ^50^; Tvrdíková (2016) ^51^; Maritz & du Toit (2018) ^52^; Sewdass & Calof (2020) ^53^; Carson et al. (2020) ^54^; Talaoui et al. (2020) ^55^; Calheiros et al (2023) ^56^; Asante et al. (2024), ^57^ state that this type of methodology allows agri-food companies to understand the global market, identify export opportunities and anticipate changes in demand.

Block 2, entitled «Strategic and Tactical Applications in the Marketing of Agricultural Products», includes authors such as Ahmed & Sallam. (2020) ^58^; Lu et al. (2019) ^59^; Kalafsky & Gress (2014) ^60^; Kalafsky (2009) ^61^; Simeon (2006) ^62^; Gumede (2004) ^63^; Zaharieva et al. (2004) ^64^; Gumede & Rasmussen (2002) ^65^; Hassani & Blais (2024) ^66^; Sutanto et al., (2024) ^67^; Nurudin (2023) ^68^; Junla & Naipinit (2024) ^69^; Borrero (2023) ^70^; Johns et al. (2024) ^71^; Tiwari et al. (2023) ^72^; De Silva et al. (2021) ^73^; Osano (2019) ^74^; Sundström et al. (2021) ^75^; Trienekens et al. (2018) ^76^; Ibeh et al. (2006) ^77^; Mashahadi et al. (2016) ^78^; Lemos & Porto (1998) ^79^; Paap (2020) ^80^; Sundström et al. (2020) ^81^; Elsharnouby et al. (2024) ^82^; Wang et al. (2024) ^83^; Munawar et al. (2024) ^84^; González García et al. (2023) ^85^; Neethirajan (2023), ^86^ explain that its use allows supply to be adapted to international demand, improves logistics processes and strengthens the competitiveness of agro-exportable products.

In Block 3, «Impact, Effectiveness and Convergences in Empirical Results», authors Pham & Petersen (2021) ^87^; Hashim et al. (2020) ^88^; Katsikea et al. (2019) ^89^; Yan et al. (2017) ^90^; Pascucci et al. (2016) ^91^; Navarro et al. (2016) ^92^; Chung (2012) ^93^; Doole et al. (2006) ^94^; Toften (2005) ^95^; Gómez-Prado et al. (2022) ^96^; Ngo (2023) ^97^; Niwash et al. (2022) ^98^; Alfarajat (2023) ^99^; Fakhreddin & Foroudi (2022) ^100^; Robson et al. (2023) ^101^; Kovács & Szakály (2022) ^102^; Rifqi et al. (2024) ^103^; Bao (2020) ^104^; Falahat et al. (2020) ^105^; Helm et al. (2020) ^106^; Joensuu et al. (2018) ^107^; Usman et al. (2020) ^108^; Bertello et al. (2021) ^109^; Dzogbenuku & Keel-son (2019) ^110^; Tarek et al. (2016) ^111^; Tanev & Bailetti (2008) ^112^; Story et al. (2015) ^113^; Baker & Sinkula (2005) ^114^; Zulkiffli & Perera (2012) ^115^; Homburg et al. (2017) ^116^; Caseiro & Coelho (2019) ^117^; Bicakcioglu & Ipek (2020) ^118^; Joshi et al. (2024) ^119^; Isichei et al. (2023), ^120^ mentioned that its effectiveness is based on the ability to transform information into strategic decisions that optimise adaptation to international markets, consolidate customer orientation and increase profitability.

## 4. Discussion

The scientific literature on MI has expanded notably in recent years, with a marked acceleration from 2018 onwards. This increase reflects a growing academic interest in the management of strategic information in increasingly competitive and dynamic business environments. ^121^ As the Ref. ( 122), says, the results of this review show that systematically collected and analysed data is an important tool for making decisions in international business. The increase in publications also indicates that MI is transitioning from a primarily operational support role to a strategic function and a possible source of competitive advantage.

The bibliometric analysis revealed that the majority of scientific publications are produced by countries such as the United States, China, and the United Kingdom, whereas regions like Latin America exhibit minimal participation. This pattern aligns with the assertion in, ^123^ which links international competitiveness to a nation’s ability to generate, assimilate, and leverage strategic knowledge. Latin American countries have a strong focus on exports, especially in the agri-food sectors, but they don’t do much scientific research. This shows that there is a gap between what researchers do and what businesses do. This discrepancy may be attributable to methodological and funding limitations, insufficient investment in applied research and development, and inadequate connections among universities, public agencies, and productive sectors. ^124^

From a thematic perspective, the semantic maps and conceptual blocks identified in this study indicate three broad areas of work: (i) MI as a system for generating strategic information; (ii) MI as support for decision-making related to positioning, segmentation and logistics in international markets; and (iii) MI as a factor associated with export performance and competitiveness. The reviewed studies converge on the idea that MI reduces information asymmetries, facilitates anticipation of changes in demand and supports the internationalisation of firms, particularly SMEs. ^125^ At the same time, the increasing use of digital tools like data analytics, dashboards, and information systems shows that MI is becoming more and more a part of larger processes of digital transformation and innovation in agri-food value chains.

Even with this progress in ideas and methods, the results show that there are still some gaps. First, theoretical fragmentation continues: there is no widely accepted integrative model elucidating the interaction between MI, business strategy, innovation capabilities, and global competitiveness. Research often utilises frameworks such as market orientation, competitive advantage, or resource-based theories; however, they seldom position MI as a central, overarching construct. Second, the literature’s coverage of sectors is still limited. Most empirical research concentrates on industrial and service sectors; there is a paucity of studies investigating productive sectors like agro-exports, despite agriculture’s heightened susceptibility to trade uncertainty, climate variability, and regulatory shocks, along with its significant reliance on strategic information for global market competition. ^126^ Third, while some contributions address issues of sustainability, quality and traceability, these dimensions are often treated as secondary outcomes rather than as integral components of MI systems.

This study adds to the body of research in a number of ways. It organises what we already know about MI in international marketing and shows how MI can help agricultural products go global by helping companies make better decisions about exports, manage risk better, and be more responsive to changes in the market. By using Bradford’s and Lotka’s laws to compare three big databases, the study gives a complete picture of where research is focused in terms of geography, institutions, and themes. Furthermore, the delineation of three conceptual blocks elucidates the interconnections among theoretical foundations, strategic and tactical applications, and empirical evidence regarding impact, thus providing a structured framework for interpreting the field.

The results also point to certain areas where more research is needed. One priority is to adapt and test MI models in agro-export chains, including variables like quality, traceability, certification, and sustainability, which are becoming more important in international markets. Another goal is to create indicators and commercial information systems for emerging economies that are specific to each sector. This will be done by combining bibliometric and qualitative methods with primary data from businesses, cooperatives, and producer organisations. These studies could help bring together the ideas behind MI and the real-world needs of people who export agricultural goods in places like Latin America.

There are some problems with this study. It depends on three main indexed databases, which might not include important work that was published in local or non-indexed journals or in grey literature. But this choice of method also makes sure that the corpus being studied is very scientifically rigorous and can be compared to other studies. Future reviews might look for more information in regional repositories and use other methods, like meta-analyses or mixed-method designs, to get a better idea of how MI works in certain agri-food settings. In general, the evidence presented here shows both the progress that has been made and the problems that still need to be solved in making MI a strategic tool for long-term competitiveness in international agricultural trade.

## 5. Conclusions

This review demonstrates that MI is an effective tool for enhancing the international marketing of agricultural products. The analysis of 83 scientific articles reveals that MI correlates with enhanced export performance by diminishing uncertainty, forecasting market trends, and facilitating more informed decision-making within exporting enterprises. However, the use of MI in agro-export chains is still limited and uneven, especially in developing countries. This shows that there is a lot of room for improvement.

With regard to the first specific objective, the findings suggest that the relationship between MI and agricultural trade is still at an early stage of theoretical consolidation. Although the literature broadly agrees that MI can generate competitive advantage, there is conceptual fragmentation and a lack of integrative studies that explicitly situate MI within the specific dynamics of agri-food and agro-export sectors.

In relation to the second specific objective, the reviewed studies show that MI is linked to the marketing of agricultural products in foreign markets through the provision of information on international prices, consumer preferences, trade barriers and segmentation strategies. At the same time, most contributions emphasise that its impact is greater when information is systematically transformed into strategic actions within the exporting firm. This requires institutional capacities to develop and maintain commercial information systems, as well as organisational routines for analysing and using data in export planning.

For the third specific objective, there is a lot of evidence that MI improves export performance when it is used in a planned and organised way. Its success depends on having access to reliable information and the company’s ability to understand and use that information in business decisions. This theoretical and empirical consensus substantiates the perspective that MI ought to be regarded as a strategic export management practice rather than a mere data collection exercise.

To summarize, market intelligence is a strategic resource for strengthening the international competitiveness of agro-exporting firms; however, its adoption remains constrained by barriers that are particularly significant in developing countries. At the technological level, the main limitations are associated with the insufficient integration of digital tools, analytical platforms, and sectoral information systems capable of transforming dispersed data into timely commercial intelligence. At the organizational level, limited expertise in strategic analysis and the absence of systematic routines for interpreting and using information restrict the ability of many small and medium-sized enterprises to incorporate MI into export planning. These constraints are compounded by institutional barriers, including insufficient investment in applied research and development and weak linkages among universities, public agencies, and productive sectors, which hinder the generation, transfer, and effective use of specialized information for international markets. In this context, strengthening MI in developing agro-export economies requires sector-specific models adapted to their technological and institutional capabilities and capable of integrating commercial information, organizational knowledge, and decision-making processes oriented toward international markets.

Future research must transcend descriptive methodologies and quantify the influence of market intelligence on particular business outcomes, including export volume, market diversification, access to certifications, and positioning within higher-value segments. It would also be relevant to explore how MI can be integrated with technological tools and agricultural information platforms, including big data analytics and digital dashboards. In parallel, there is a need to design contextualised methodological frameworks that strengthen both the operational and strategic dimensions of agri-food value chains, connecting MI not only to competitiveness, but also to broader sustainability objectives.

### Ethics and consent

Ethics and Consent were not required for the performed study.

## Data availability

### Underlying data

Zenodo: Market intelligence for exporting agricultural products to foreign markets: A PRISMA-based bibliometric and systematic review. Version 1. https://doi.org/10.5281/zenodo.17556196 (Altamirano and Morán, 2025). ^127^

The project contains the following underlying data:

• General data on the RSL process.xlsx (Results of the systematic review of the literature from Scopus, Web of Science, and the Dimensions database).
• PRISMA 2020 checklist.

### Extended data

Zenodo: Market intelligence for exporting agricultural products to foreign markets: A PRISMA-based bibliometric and systematic review. Version 1. https://doi.org/10.5281/zenodo.17556196 (Altamirano and Morán, 2025). ^127^

The project contains the extended data data:

Figure 1. Prisma 2020 flow chart for selecting and including systematic review documents.

Figure 2. Weighted bar charts of the distribution of bias risk judgements within each bias domain.

Figure 3. Traffic light charts of domain-level judgements for each individual result.

Figure 4. Percentage of total publications on market intelligence and marketing identified in Scopus, Dimensions, and WoS between 1960 and 2024.

Figure 5. Trends in publications on the growth of market intelligence in marketing between 1960 and 2024.

Figure 6. Main authors contributing to the collection, obtained from the Scopus database.

Figure 7. Main authors contributing to the collection, obtained from the DIMENSIONS database.

Figure 8. Main authors contributing to the collection, obtained from the Web of Science database.

Figure 9. Publications by country on market intelligence in marketing, obtained from the Scopus database.

Figure 10. Publications by country on market intelligence in marketing, obtained from the Dimensions database.

Figure 11. Publications by country on market intelligence in marketing, obtained from the Web of Science database.

Figure 12. Semantic map of the relationship between market intelligence and marketing, obtained from the open-source programme VOSviewer, with metadata from Scopus.

Figure 13. Semantic map of the relationship between market intelligence and marketing, obtained from the open-source programme VOSviewer, with metadata from Dimensions.

Figure 14. Semantic map of the relationship between market intelligence and marketing, obtained from the open-source programme VOSviewer, with metadata from WOS.

**Free access to RSL data:** Zenodo: Exploring the use of market intelligence for marketing agri-cultural products to foreign markets from the perspective of bibliometric analysis with Prisma. Version 1. https://doi.org/10.5281/zenodo.17556196 (Altamirano and Morán, 2025). ^127^

The data is available under the terms of the Creative Commons Zero v1.0 Universal (CC0 1.0) licence.
