# Supplementary material for: Market intelligence for exporting agricultural products to foreign markets: A PRISMA-based bibliometric and systematic review
Source: F1000Res. 2026 Aug 21;15:316. Originally published 2026 Feb 24. [Version 2] doi: 10.12688/f1000research.175062.2 (PMC13579032; doi:10.12688/f1000research.175062.2)
Supplement: Peer review history

# Reviewer report: Reviewer response for version 2

**DOI:** 10.5256/f1000research.208573.r520853

**Published:** 2026-09-25

**Authors:** Nebi Seren

**Affiliations:** 1 Bursa Uludağ Üniversitesi, Orhangazi Yeniköy Asil Çelik Meslek Yüksekokulu, Bilgisayar Teknolojileri Bölümü, Bursa, Turkey

I have reviewed Version 2 of this article, which presents a PRISMA-based bibliometric and systematic review of market intelligence in the international marketing of agricultural products. The rationale and objectives are clear. Table 1 reports database-specific search strings, and the main analytical steps are described clearly.

- I noted several minor reporting issues. The description of the study as “cross-sectional, descriptive and non-experimental” is commonly associated with empirical survey designs. “A PRISMA-based bibliometric and systematic literature review” would describe this design more accurately. On page 16, “a causal line” should be replaced with “a conceptual sequence”, since keyword co-occurrence indicates association rather than causality.
- Two internal count discrepancies should also be corrected. On pages 6–7, 1,454 records screened minus 1,044 excluded leaves 410, while the next stage reports 393 reports sought. Section 2.2.1 reports 1,465 records after initial refinement and 1,454 after deduplication, while Figure 4 on page 9 is based on 1,463 publications. The authors should identify the stage represented by Figure 4 and align the totals.

These points concern terminology and internal numerical consistency. They do not change my assessment of the review design or its principal conclusions. I consider the article to be of an acceptable scientific standard and recommend approval.

Are the rationale for, and objectives of, the Systematic Review clearly stated?

Yes

Is the statistical analysis and its interpretation appropriate?

Yes

If this is a Living Systematic Review, is the ‘living’ method appropriate and is the search schedule clearly defined and justified? (‘Living Systematic Review’ or a variation of this term should be included in the title.)

Not applicable

Are sufficient details of the methods and analysis provided to allow replication by others?

Yes

Are the conclusions drawn adequately supported by the results presented in the review?

Yes

Reviewer Expertise:

Business and Management; International Marketing; Market Intelligence; Bibliometrics; Systematic Literature Reviews; Data Science

I confirm that I have read this submission and believe that I have an appropriate level of expertise to confirm that it is of an acceptable scientific standard.

# Reviewer report: Reviewer response for version 2

**DOI:** 10.5256/f1000research.208573.r515052

**Published:** 2026-09-15

**Authors:** Giang-Do Nguyen

**Affiliations:** 1 Nguyen Tat Thanh University, Ho Chi Minh City, Vietnam

Generally, I recommend **Acceptable**.

 The remaining issues mainly concern

 (1) language polishing and 

 (2) the manuscript describes the design as “cross-sectional, descriptive and non-experimental,” which is more typical of empirical survey studies and should be replaced with clearer terminology such as “PRISMA-informed bibliometric and systematic literature review.”

 (3) The authors consider presenting the exact search strings, fields searched, and filters applied.

Are the rationale for, and objectives of, the Systematic Review clearly stated?

Yes

Is the statistical analysis and its interpretation appropriate?

Yes

If this is a Living Systematic Review, is the ‘living’ method appropriate and is the search schedule clearly defined and justified? (‘Living Systematic Review’ or a variation of this term should be included in the title.)

Yes

Are sufficient details of the methods and analysis provided to allow replication by others?

Yes

Are the conclusions drawn adequately supported by the results presented in the review?

Yes

Reviewer Expertise:

Business and Management; International marketing; Human-Technology Interaction;

I confirm that I have read this submission and believe that I have an appropriate level of expertise to confirm that it is of an acceptable scientific standard.

# Reviewer report: Reviewer response for version 2

**DOI:** 10.5256/f1000research.208573.r513404

**Published:** 2026-08-26

**Authors:** Luis Eduardo Solis Granda

**Affiliations:** 1 Universidad Estatal de Milagro, Milagro, Ecuador

I have carefully reviewed the revised version of this manuscript, together with the authors' responses to the comments raised in my previous report. The authors have made a genuine effort to address each of the points I raised, and the revisions have improved the clarity and rigor of the study.

 In particular, the introduction and research questions are now presented more clearly, and the methodological description of the bibliometric and systematic review approach is easier to follow. The connection between the theoretical framework on market intelligence and the specific context of agricultural exports is also better justified.

 I have no further substantive comments to make. I consider that the article meets the necessary standards for indexing and can be approved in its current form.

Are the rationale for, and objectives of, the Systematic Review clearly stated?

Yes

Is the statistical analysis and its interpretation appropriate?

Partly

If this is a Living Systematic Review, is the ‘living’ method appropriate and is the search schedule clearly defined and justified? (‘Living Systematic Review’ or a variation of this term should be included in the title.)

Not applicable

Are sufficient details of the methods and analysis provided to allow replication by others?

Partly

Are the conclusions drawn adequately supported by the results presented in the review?

Partly

Reviewer Expertise:

Business and Management is the primary area, as it corresponds to the journal's classification and the study's core focus on market intelligence as a strategic management tool. Agricultural Economics reflects the sectoral context of the review. Information and Library Science covers the bibliometric methodology applied. International Trade and International Business aligns with the export performance dimension that runs throughout the paper.

I confirm that I have read this submission and believe that I have an appropriate level of expertise to confirm that it is of an acceptable scientific standard.

# Reviewer report: Reviewer response for version 1

**DOI:** 10.5256/f1000research.193018.r493504

**Published:** 2026-06-23

**Authors:** Zool Hilmi Ismail

**Affiliations:** 1 Center for Artificial Intelligence and Robotics, University of Technology Malaysia, Kuala Lumpur, Kuala Lumpur, Malaysia

This paper presents a PRISMA 2020-based bibliometric and systematic review of MI in the international marketing of agricultural products, analyzing 83 eligible articles from Scopus, Dimensions, and WoS. The study is well-structured and addresses a relevant gap in the literature, particularly for Latin American agro-export economies. However, several methodological inconsistencies, figure labelling errors, and gaps in synthesis must be resolved before the paper can be considered fully sound.

 My comments and suggestions are as follows:

 1. Fig. 1 (pg. 7) labels all three identification sources as "Scopus (n = 99)", "Scopus (n = 39,680)", and "Scopus (n = 188)", when they should read Scopus, Dimensions, and WoS respectively. This must be corrected.

 2. Table 1 (pg. 5) records the Dimensions search date as 21 November 2023 while Scopus and WoS were searched on 20 September 2025, a near two-year gap? Please justify. 

 3. Fig. 7 (pg. 10) and Fig. 8 (pg. 11) appear to have swapped captions?

 4. Fig. 4 (pg. 9) presents the database distribution pie chart showing an initial identification counts (39,680 vs 99 vs 188) rather than the final corpus composition. Why? 

 5. The exclusion criterion "studies not relevant to the Sustainable Development Goals" (pg 5) is applied without identifying which SDGs are relevant or providing a mapping?

 6. Table 6 (pg 19) assigns several studies to Block 2 that are not clearly related to agricultural marketing. See Istanto et al. (2023) on tourism marketing and Królak et al. (2022) on fibre-enriched bread innovation. 

 7. In the same table, Wagner et al. (2002), is assigned to Block 1 on MI fundamentals in agricultural exports, but the study is about immigration and the trade of Canadian provinces. Suggest removing it.

 8. Pg 20, "a structured framework for interpreting the fiel"; "fiel" should read "field."

Are the rationale for, and objectives of, the Systematic Review clearly stated?

Yes

Is the statistical analysis and its interpretation appropriate?

Partly

If this is a Living Systematic Review, is the ‘living’ method appropriate and is the search schedule clearly defined and justified? (‘Living Systematic Review’ or a variation of this term should be included in the title.)

Yes

Are sufficient details of the methods and analysis provided to allow replication by others?

Yes

Are the conclusions drawn adequately supported by the results presented in the review?

Yes

Reviewer Expertise:

Automated system, AI Forecasting

I confirm that I have read this submission and believe that I have an appropriate level of expertise to confirm that it is of an acceptable scientific standard, however I have significant reservations, as outlined above.

# Reviewer report: Reviewer response for version 1

**DOI:** 10.5256/f1000research.193018.r468084

**Published:** 2026-03-26

**Authors:** Luis Eduardo Solis Granda

**Affiliations:** 1 Universidad Estatal de Milagro, Milagro, Ecuador

**Summary of the Article and Relevance**

 The article by Altamirano-Gonzales and Morán-Santamaría provides a comprehensive systematic and bibliometric review of Market Intelligence (MI) within the global agricultural sector. By utilizing the PRISMA 2020 framework and analyzing 83 eligible articles from Scopus, Dimensions, and Web of Science, the study successfully maps the evolution of MI as a strategic tool. Its relevance is particularly high given the need for data-driven decision-making in agro-exports, especially for regions like Latin America that show a gap in scientific production despite their market importance.

**Strengths**

- **Methodological Rigor:** The combination of three major databases and the use of specialized software (VOSviewer and Bibliometrix) ensures a robust bibliometric mapping.
- **Thematic Structure:** The organization of the qualitative synthesis into three conceptual blocks (fundamentals, strategic applications, and empirical impact) is logical and highly informative.
- **Identification of Research Gaps:** The study effectively highlights the geographical concentration of research and the limited participation of developing economies.

**Areas for Improvement (Basis for "Partly" Ratings)**

 To move the status from "Partly" to "Yes" in the methodology and analysis sections, the following points should be addressed:

1. **Replicability (Methods):** While the use of PRISMA is clear, the authors should provide the **exact search strings** used for each database. This is essential for full scientific replication.
2. **Cluster Interpretation (Analysis):** The interpretation of the co-occurrence maps is currently descriptive. I recommend that the authors further explain the *interconnectivity* between the identified clústers to show how theoretical concepts link to practical agro-exporting strategies.
3. **Discussion Depth (Conclusions):** The conclusion regarding limited adoption in developing countries is well-founded but would benefit from a more detailed discussion on the specific technological or institutional barriers that hinder MI implementation in these regions.

**Final Status Confirmation**

 I confirm my status as **Approved with Reservations**. I believe the article is of an acceptable scientific standard and offers a significant contribution, but it requires these specific revisions to be considered fully scientifically sound.

Are the rationale for, and objectives of, the Systematic Review clearly stated?

Yes

Is the statistical analysis and its interpretation appropriate?

Partly

If this is a Living Systematic Review, is the ‘living’ method appropriate and is the search schedule clearly defined and justified? (‘Living Systematic Review’ or a variation of this term should be included in the title.)

Not applicable

Are sufficient details of the methods and analysis provided to allow replication by others?

Partly

Are the conclusions drawn adequately supported by the results presented in the review?

Partly

Reviewer Expertise:

Business and Management is the primary area, as it corresponds to the journal's classification and the study's core focus on market intelligence as a strategic management tool. Agricultural Economics reflects the sectoral context of the review. Information and Library Science covers the bibliometric methodology applied. International Trade and International Business aligns with the export performance dimension that runs throughout the paper.

I confirm that I have read this submission and believe that I have an appropriate level of expertise to confirm that it is of an acceptable scientific standard, however I have significant reservations, as outlined above.
